# Enhanced two-dimensional ferromagnetism in van der Waals β-UTe_3_ monolayers

**DOI:** 10.1126/sciadv.aea6436

**Published:** 2026-03-25

**Authors:** Sean M. Thomas, Andres E. Llacsahuanga Allcca, Wolfgang Simeth, Caitlin S. Kengle, Zachary W. Riedel, Fabio Orlandi, Dmitry Khalyavin, Pascal Manuel, Filip Ronning, Eric D. Bauer, Joe D. Thompson, Jian-Xin Zhu, Allen O. Scheie, Yong P. Chen, Priscila F. S. Rosa

**Affiliations:** ^1^ Los Alamos National Laboratory, Los Alamos, NM 87545, USA.; ^2^ Department of Physics and Astronomy, Purdue University, West Lafayette, IN 47907, USA.; ^3^ ISIS Facility, Rutherford Appleton Laboratory, Chilton, Didcot OX11 0QX, UK.; ^4^ School of Electrical and Computer Engineering, Purdue University, West Lafayette, IN 47907, USA.; ^5^ Purdue Quantum Science and Engineering Institute, Purdue University, West Lafayette, IN 47907, USA.; ^6^ Institute of Physics and Astronomy and Villum Centers for Dirac Materials and for Hybrid Quantum Materials, Aarhus University, 8000 Aarhus-C, Denmark.; ^7^ WPI Advanced Institute for Materials Research (AIMR), Tohoku University, Sendai 980-8577, Japan.

## Abstract

The discovery of local-moment magnetism in van der Waals (vdW) semiconductors down to the single-layer limit has led to a paradigm shift in the understanding of two-dimensional (2D) magnets. The incorporation of strong electronic and magnetic correlations in 2D vdW metals remains a sought-after platform to enable control of emergent quantum phases and to achieve more theoretically tractable microscopic models of complex materials. To date, however, there is limited success in the discovery of such metallic vdW platforms, and f-electron monolayers remain out of reach. Here, we demonstrate that strongly correlated β–uranium tritelluride (β-UTe_3_) can be exfoliated to the monolayer limit. Unexpectedly, β-UTe_3_ remains ferromagnetic in this limit with an enhanced ordering temperature of 35 kelvin, a factor of two larger than its bulk counterpart. Our work establishes β-UTe_3_ as a materials platform for investigating and modeling correlated behavior in the monolayer limit and opens numerous avenues for quantum control with, e.g., strain engineering.

## Teaser

Van der Waals β-UTe_3_ is a ferromagnetic materials platform for investigating correlated behavior in the monolayer limit.

## INTRODUCTION

Two decades ago, the exfoliation of graphite down to single-layer graphene sheets transformed the aspiration of atomic-scale materials design into reality (*1*). When two or more graphene layers are stacked, novel quantum states can be designed from strongly correlated electrons that were not present in a single layer (*2*). Examples include fractional Chern insulator states in twisted bilayer graphene (*3*) and unconventional, chiral superconductivity in pentalayers (*4*). Since graphene, many atomically thin, layered van der Waals (vdW) crystals have been found and now provide a vast materials platform for exceptional properties and applications in two dimensions (2D) (*5*–*7*). A notable recent development is the observation of local-moment magnetism in semiconducting monolayers (*8*–*13*). Although conventional wisdom does not expect ordering in the isotropic 2D limit due to the Mermin-Wagner theorem (*14*), the presence of magnetic anisotropy and finite-size effects enables the stabilization of both ferromagnetic (FM) and antiferromagnetic (AFM) states (*8*–*13*, *15*).

In a metallic 2D magnet, enhanced quantum fluctuations are expected, and magnetic order may be destabilized in the 2D limit. As a result, sought-after states of matter may emerge in the monolayer limit ranging from a 2D strange metal that defies the concept of quasiparticles within Fermi-liquid theory (*16*, *17*) to unconventional superconductivity with topological excitations envisioned as the building block of quantum computing (*18*).

Most known vdW magnets to date, however, are semiconducting and exhibit rather robust magnetic order with sizable ordered moments. Rare examples of metallic vdW magnets include d-electron ferromagnets Fe*_n_*GeTe_2_ (n=3,4,5) and f-electron antiferromagnets CeSiI, UOTe, and *R*Te_3_ (*R* = rare earth). UOTe orders at 150 K with a small electronic (Sommerfeld) contribution to the specific heat, γ=6 mJ/mol·K^2^, which indicates weak hybridization between local 5f moments and conduction electrons (*19*). *R*Te_3_ members also show local-moment magnetism (*20*, *21*), and GdTe_3_ has been exfoliated only to the few-layer regime (*22*). Although Fe*_n_*GeTe_2_ (*23*–*28*) and CeSiI (*29*) do show signs of modest electronic correlations, as evident from their moderately enhanced Sommerfeld coefficients, these systems appear to be far from a magnetic instability. In addition, UOTe and CeSiI have not been exfoliated down to the monolayer limit.

Here, we investigate actinide vdW β-UTe_3_ down to the half–unit-cell limit. Bulk crystals show easy-axis magnetic anisotropy and an FM transition at $T_{C}=15$ K in magnetic susceptibility data. Our specific heat and neutron diffraction measurements are consistent with a 3D FM order wherein the interlayer magnetic exchange interaction is much smaller than the intralayer interaction, typical of quasi-2D magnetic systems (*30*, *31*). 2D correlations are observed above $T_{C}$ in neutron diffraction experiments. A sizable Sommerfeld coefficient of γ=130 mJ/mol·K^2^ is observed in β-UTe_3_, akin to other strongly correlated 5f Kondo metals and superconductors (*32*–*35*). β-UTe_3_ remains FM down to the half–unit-cell limit with an enhanced ordering temperature of $T_{C}^{\text{ML}}=35$ K, which stems from an “extraordinary” phase transition. Our work establishes β-UTe_3_ as a materials platform for investigating, controlling, and modeling the interplay between dimensional confinement, electronic correlations, and magnetism in 2D vdW materials.

## RESULTS

**Fig. 1.**
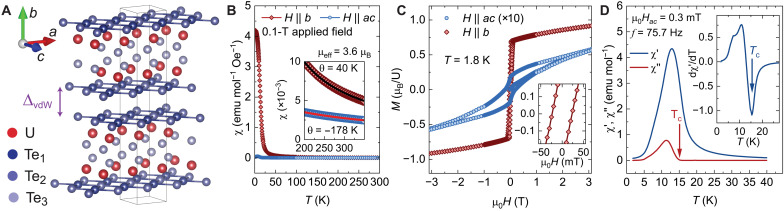
Crystal structure and magnetic measurements. (**A**) vdW crystal structure of β-UTe_3_. (**B**) Magnetic susceptibility versus temperature for fields applied along the *b* axis and in the *ac* plane. The inset shows Curie-Weiss (CW) fits between 200 and 350 K (solid lines). emu, electromagnetic unit. (**C**) Magnetization versus field at 1.8 K. The *ac* plane data are multiplied by 10 for clarity. The inset shows a zoomed-in view of the *b*-axis data. (**D**) Real ($\chi^{\prime }$) and imaginary ($\chi^{\prime \prime }$) parts of the ac susceptibility versus temperature. Inset shows the derivative of $\chi^{\prime }$ with respect to temperature.

β-UTe_3_ crystallizes in space group Cmcm (no. 63) in a pseudotetragonal structure with lattice parameters a = c = 4.338 Å and b = 24.743 Å (*36*). The layered crystal structure, shown in Fig. 1A, reveals a vdW gap ($\Delta_{\text{vdW}}$) between the square nets of Te_1_ and Te_2_ atoms. The position of $\Delta_{\text{vdW}}$ suggests the possibility of exfoliation down to a thickness of half of a unit cell (UC), which is indeed consistent with the results presented below. Each half UC is comprised of two layers of uranium square nets with AB stacking. Uranium atoms experience an environment that is locally noncentrosymmetric, although the UC has an inversion center that lies in the vdW gap.

### 3D FM order in bulk β-UTe_3_

Magnetic susceptibility measurements, presented in Fig. 1B, reveal a highly anisotropic response and a clear FM transition at low temperatures, in agreement with previous reports (*36*). For out-of-plane fields, a Curie-Weiss (CW) fit yields a positive Weiss temperature, $\theta_{W}=40$ K, consistent with interlayer FM interactions (inset of Fig. 1B). Similar fits to the ac-plane data yield a larger negative value for $\theta_{W}$. This sign, in principle, suggests the presence of competing intralayer AFM interactions. These values should be taken with caution, however, because crystal field effects are expected to be relevant here. The low-temperature magnetic exchange interactions may be substantially different compared to these high-temperature fits. Our CW fit also yields an effective moment of $\mu_{\text{eff}}=3.6\;\mu_{B}$, which matches the expected Hund’s values for either U^3+^ (3.62 $\mu_{B}$) or U^4+^ (3.58 $\mu_{B}$). Crystal-field fits to the magnetic susceptibility data did not provide a definitive determination of the uranium oxidation state due to overparameterization, and an accurate determination of the crystal field scheme for β-UTe_3_ will therefore require more direct spectroscopic measurements (*37*). Overall, our magnetic susceptibility data reveal that the easy magnetization axis is the *b* axis, which, in turn, shows a tiny coercive field of less than 100 mT in magnetization loops (Fig. 1C), consistent with soft FM. To precisely determine the critical FM temperature, $T_{C}$, we performed ac susceptibility measurements at low driving fields (*38*). As shown in Fig. 1D, $\chi^{\prime \prime }$ sets in at $T_{C}=15$ K, consistent with the minimum in $d\chi^{\prime }/\mathrm{dT}$ (inset).

According to the Ginzburg-Landau mean-field theory, the magnetization squared ($M^{2}$) is linearly proportional to $H_{\text{int}}/M$, where $H_{\text{int}}$ is the applied field after taking into account demagnetization effects (*39*). An $M^{2}$ versus $H_{\text{int}}/M$ plot, known as the Arrott plot (*40*), should, in turn, yield linear curves close to $T_{C}$, consistent with mean-field critical exponents β=0.5 and γ=1.0. The substantial curvature found in fig. S7 readily reveals that the FM transition in β-UTe_3_ is not mean field, and different exponents are at play. Efforts to obtain critical exponents using a modified Arrott plot, however, did not yield self-consistent results (*41*).

**Fig. 2.**
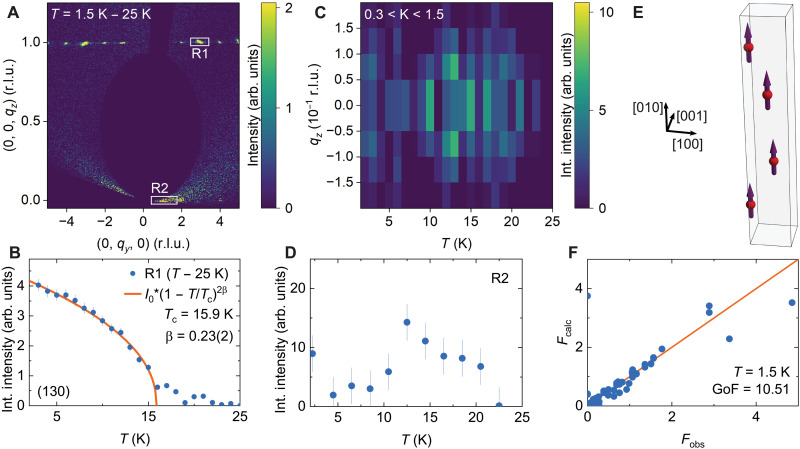
Neutron diffraction study of bulk magnetic order. (**A**) Momentum-space slices through magnetic diffraction intensity obtained by temperature subtraction (1.5 to 25 K). Color coding reflects scattering intensity in the plane $q=\left(0,q_{y},q_{z}\right)$, which is a superposition of two twins with swapped H and L axes: (0, *K*, *L*) and (*H*, *K*, 0) planes. Peaks on the line $q_{z}=1$ correspond to resolution-limited magnetic Bragg peaks (see section S11). The two rectangles R1 and R2 indicate schematically integration windows for the integrated intensities presented in (B) and (D). arb. units, arbitrary units. (**B**) Thermal variation of the magnetic Bragg peak Q=(130). Blue data points denote integrated intensity of the Bragg peak in the rectangular region of interest R1. The orange line corresponds to an order parameter fit with the indicated parameters. Int., Integrated. (**C**) Variation of diffuse scattering on the K axis in region R2 as a function of momentum space coordinate $q_{z}$ and temperature. Intensity was integrated within a momentum-space volume of 0.1 reciprocal lattice units (r.l.u.) along $q_{z}$ and $q_{x}$. For illustration purposes, data were symmetrized with a reflection around $q_{z}=0$. (**D**) Temperature dependence of total integrated diffuse scattering intensity (blue data points) integrated over 0.3≤K≤1.5, $-0.05\leq q_{x}\leq 0.05$, and $-0.07\leq q_{z}\leq 0.13$. Measurements were performed at the same temperatures as in (C) and averaged over pairs of temperatures. (**E**) Ferromagnetic spin texture determined from magnetic structure refinements and a careful analysis of recorded magnetic structure factor at T=1.5 K (see section S11). The moments are essentially pointing along the *b* axis. (**F**) Comparison of calculated and observed structure factor for the combined magnetic and structural refinement carried out at T=1.5 K. The refined magnetic moment obtained from this fit is $M=0.48\left(11\right)\;\mu_{B}$. GoF, goodness of fit.

To address this challenge, we turn to neutron diffraction measurements of a bulk crystal. The temperature-subtracted magnetic neutron diffraction intensity map, shown in Fig. 2A, reveals Bragg peaks at integer valued momentum transfers (*H*, *K*, *L*), consistent with 3D FM order. Figure 2B presents the temperature dependence of the integrated intensity around R1, the magnetic Bragg peak at momentum transfer (1, 3, 0). An order parameter fit (solid line) yields a critical exponent of β=0.23(2) and a critical temperature of $T_{C}=15.9\left(9\right)$ K. The extracted exponent does not match the mean-field value (β=0.5), in agreement with magnetization data. The exponent also does not match the expected values from the 3D Heisenberg (β=0.365), the 3D Ising (β=0.325), or the 2D Ising (β=0.125) models (*14*, *39*). Instead, β=0.23 is the expectation from a 2D XY phase transition in finite-sized samples (*42*). This exponent has been observed in many layered vdW Heisenberg magnets with planar anisotropy because these materials can be treated as quasi-2D XY systems (*43*, *44*). Because β-UTe_3_ displays easy-axis anisotropy with spins pointing out of the plane, a 2D Ising model seems much more likely. In the presence of long-range interactions, it has been shown that the 2D Ising model can take on exponent values continuously in the range between β=0.125 and β=0.5 depending on the interaction length scale (*45*).

Evidence for 2D magnetic correlations is observed in the diffraction results within area R2 (see Fig. 2A). Figure 2C shows a color map of the integrated intensity as a function of temperature and momentum space coordinate *L*. The corresponding temperature-subtracted diffraction intensity, shown in Fig. 2D, reveals a diffuse scattering contribution along the reciprocal *K* axis at 15 K. Notably, the diffuse signal survives well above $T_{C}$, consistent with the presence of 2D correlations in the *ac* plane that peak around $T_{C}$. This is similar to the behavior observed in CrSBr (*44*). Magnetic structure refinements reveal an FM structure with moments along the *b* axis, as shown in Fig. 2 (E and F), and a magnetic moment of M=0.48(11) $\mu_{B}$ (see section S11 for details). The moment reduction compared to the Hund’s effective moment likely stems from a combination of crystal-field effects and Kondo screening.

### Evidence for 2D magnetism, electronic correlations, and metallicity

**Fig. 3.**
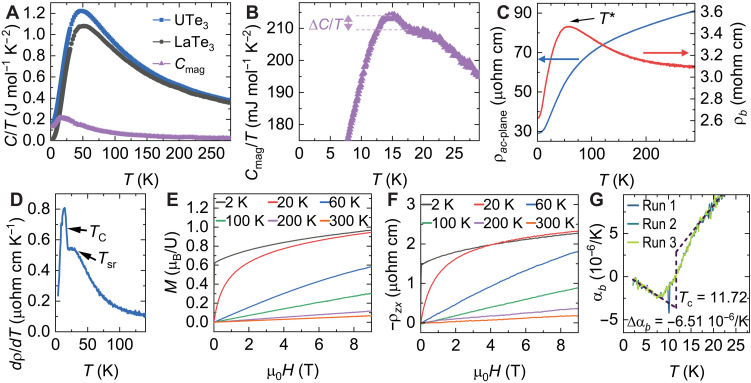
Physical property measurements. (**A**) Specific heat over temperature versus temperature for β-UTe_3_, LaTe_3_, and the difference (*C*_mag_). (**B**) A zoomed-in view of *C*_mag_/T versus temperature shows a slight feature (5 mJ/mol·K^2^) at *T*_c_. (**C**) Resistivity versus temperature. The *b* axis resistivity shows a peak near $T^{*}=60$ K. (**D**) In-plane resistivity derivative versus temperature from a second sample. The arrows indicate a jump at *T*_c_ and a broad shoulder near *T*_sr_ = 30 K, attributable to the onset of short-range magnetic correlations. (**E**) Magnetization versus field applied along the *b* axis. (**F**) Negative of Hall resistivity versus field applied along the *b* axis. (**G**) Thermal expansion coefficient along the *b* axis.

Our thermodynamic data point to a highly correlated, 2D magnetic state in β-UTe_3_. Figure 3A shows the temperature-dependent specific heat, C/T, for both β-UTe_3_ and its nonmagnetic analog LaTe_3_, which is used as the phonon reference. Notably, C/T data lack the expected λ-like peak characteristic of a 3D second-order phase transition. The magnetic contribution to the specific heat, $C_{\text{mag}}/T$, increases on cooling as magnetic entropy builds up. At $T_{C}$, however, only a tiny anomaly is observed, whose entropy is about 15 mJ/mol·K or 0.3% of Rln2, the entropy of a doublet ground state. A ground-state Kramer’s doublet is expected in the case of U^3+^ valence. For U^4+^, a ground-state quasidoublet is usually necessary for the existence of a finite magnetic moment, although other more exotic mechanisms have been proposed (*46*). The entropy reaches Rln2 only at 30 K, consistent with the presence of both short-range 2D correlations above $T_{C}$, as observed in neutron diffraction, and Kondo hybridization. The small anomaly at $T_{C}$ is reminiscent of cuprate antiferromagnet La_2_CuO_4_, which shows no detectable entropy around its AFM transition at $T_{N}=304$ K (*31*). This apparent contradiction can be solved by recalling that the entropy of a 3D transition in a layered material will depend on the strength of the interlayer exchange interaction, $J_{⊥}$. In La_2_CuO_4_, $J_{⊥}$ is about five orders of magnitude smaller than the intralayer exchange interaction, J, and the expected entropy is below the detection limit of typical calorimeters (*30*, *31*). We thus expect that $J_{⊥}$ is a few orders of magnitude smaller than J in β-UTe_3_.

Specific heat also allows the extraction of the Sommerfeld coefficient (γ) from the residual intercept in a C/T vs $T^{2}$ plot (see fig. S4). Within Fermi-liquid theory, γ is proportional to the effective mass of electrons ($m^{*}$), and an enhanced γ reflects strong underlying electronic correlations. In β-UTe_3_, we find γ=130 mJ/mol·K^2^, akin to other strongly correlated 5f Kondo metals and superconductors (*32*–*35*). As a comparison, γ is only 0.6 mJ/mol·K^2^ in nonmagnetic LaTe_3_, consistent with an uncorrelated metal. Our results therefore point to a two–order-of-magnitude renormalization of $m^{*}$ in β-UTe_3_.

To confirm the metallic state of β-UTe_3_, we turn to electrical transport measurements and density functional theory (DFT) calculations. The temperature-dependent in-plane electrical resistivity, $\rho_{\mathrm{ac}}$(*T*), of a bulk β-UTe_3_ crystal, shown in Fig. 3C, reveals a monotonic decrease in $\rho_{\mathrm{ac}}$ on cooling, consistent with metallic behavior (red curve). In comparison, the out-of-plane resistivity, $\rho_{b}$(*T*), shows a peak near $T^{*}=60$ K. Below $T^{*}=60$ K, $\rho_{\mathrm{ac}}$ also decreases more rapidly. This is likely due to a reduction in magnetic scattering that is typical of the formation of a Kondo coherent state (*47*). At low temperatures, a small kink is observed around $T_{C}$. The transition is more visualized in the derivative of $\rho_{\mathrm{ac}}$ with respect to temperature, $d\rho_{\mathrm{ac}}/\mathrm{dT}$, shown in Fig. 3D, which reveals a peak at $T_{C}$. A small hump is also apparent at $T_{\text{sr}}=30$ K, which matches the temperature at which the specific heat entropy reaches Rln2 and marks the onset of short-range 2D magnetic correlations.

To determine the carrier density (n), we perform magnetization, M, and Hall resistivity, $\rho_{\mathrm{zx}}$, measurements with fields applied along the *b* axis, as shown in Fig. 3 (E and F), respectively. In general, $\rho_{\mathrm{zx}}$ can be written as $R_{0}H+R_{s}M$, where the first term is the ordinary Hall component due to the Lorentz force and $R_{0}$ is inversely proportional to n in a single-band approximation (*48*). Extracting $R_{0}$ from fits to the data proved challenging because the ordinary term is much smaller than the second term, $R_{s}M$, the Hall effect contribution due to the spontaneous magnetization, known as the anomalous Hall effect. $\rho_{\mathrm{zx}}$ displays a dominant anomalous component at all temperatures up to 300 K. At low temperatures, however, the nonlinearity in the anomalous contribution allows the most reliable estimate of the carrier density. At 2 K, $R_{0}=+2.6$ nΩ/T, which corresponds to a carrier density of $n∼10^{23}$ holes/cm^3^, consistent with a metallic state (see section S5 for details). The effects of pressure on $T_{C}$ can be estimated via the Ehrenfest relation through a combination of thermal expansion and heat capacity measurements. The thermal expansion coefficient along the *b* axis is shown in Fig. 3G. As detailed in section S6, the negative jump in $\alpha_{b}$ at $T_{C}$ implies that $T_{C}$ should decrease at a rate of −91 K/GPa for uniaxial stress along the *b* axis.

**Fig. 4.**
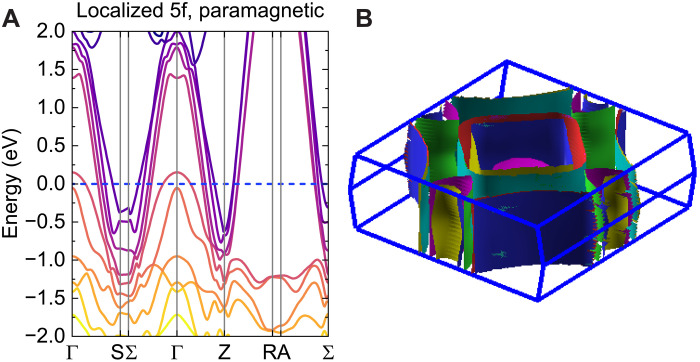
DFT calculations. (**A**) DFT band structure calculation of β-UTe_3_ in the nonmagnetic state with localized f electrons and (**B**) its corresponding Fermi surface. Spin-orbit coupling is included.

The electronic band structure of β-UTe_3_ also supports a metallic correlated state. Our DFT calculations used the generalized gradient approximation and the Perdew-Burke-Ernzerhof exchange correlational function with the WIEN2k package (*49*, *50*). DFT calculations investigated two scenarios: Figure 4A shows the band structure of β-UTe_3_ in the paramagnetic state assuming that three uranium 5f electrons are localized in the core, whereas fig. S8 shows the DFT + U band structure calculation in the FM state with a Coulomb term U=5 eV. In the localized calculation, one small hole pocket and four larger pockets from Te p bands are centered around Γ=(0,0,0), whereas four electron pockets are present around Σ=(0.258,0.258,0) and Z=(0,0,1/2). Dispersionless bands are observed along S−Σ and R−A directions, which imply a quasi-2D electronic structure along the *b* direction. Figure 4B shows the resulting quasi-2D Fermi surface of β-UTe_3_. In other tritelluride members, the tellurium 5p square nets are prone to charge-density-wave distortions (*21*). We do not, however, observe evidence for a charge-density-wave transition in β-UTe_3_. The calculated density of states at the Fermi level, $N\left(E_{F}\right)=0.56$ states/eV/f.u. (formula unit), yields γ=1.4 mJ/mol·K^2^. This small Sommerfeld coefficient, extracted in the absence of 5f electrons at $E_{F}$, is consistent with the experimentally measured coefficient for LaTe_3_ and confirms the two–order-of-magnitude renormalization of m* in β-UTe_3_.

### Enhanced magnetism in the 2D limit

**Fig. 5.**
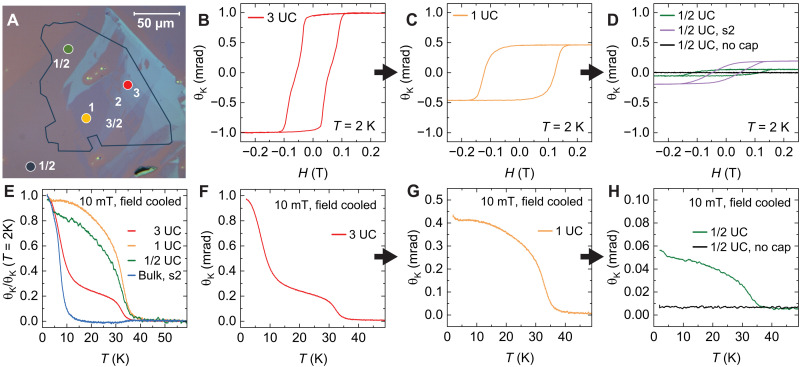
Magnetic measurements of exfoliated sample. (**A**) Optical image of exfoliated UTe_3_ before placing h-BN capping layer (indicated by gray outline). Sample s2 is not shown. (**B** to **D**) Kerr rotation versus field at 2 K for a number of different spots ranging from 3- to ^1^/_2_-UC thickness. (**E**) Normalized Kerr rotation versus temperature during cooling in a 10-mT applied field. (**F** to **H**) Kerr rotation versus temperature during cooling in a 10-mT applied field for a number of different spots.

We now turn to the evolution of the FM state down to the half–unit-cell limit. Figure 5A shows an optical image of exfoliated β-UTe_3_ flakes. The numbers indicate the thickness, in UC, of each layer and reveal successful exfoliation down to half of a UC (see section S1 for details). The colored circles correspond to the regions investigated by the polar Kerr effect (PKE).

Figure 5 (B to D) shows the polar Kerr angle, $\theta_{k}$, at 2 K as a function of fields applied along the *b* axis on a three-UC flake (Fig. 5B), a single-UC flake (Fig. 5C), and a half-UC flake (Fig. 5D). $\theta_{k}$ measures the change in the light polarization upon reflection from a magnetized material and is sensitive to out-of-plane magnetic fields. Magnetic hysteresis is observed down to the half-UC limit with a coercive field of ∼0.2 T, a value twice as large as that in bulk samples. The uncapped half-UC spot (dark blue circle in Fig. 5A) did not show a detectable Kerr signal due to flake degradation when exposed to air. FM hysteresis loops in capped flakes are also observed at 20 K, as shown in fig. S2.

To better understand the evolution of the FM state, we perform temperature-dependent PKE measurements. The flakes are cooled in a *b*-axis field of 10 mT, and measurements are performed under zero-field warming conditions. Figure 5 (F to H) shows $\theta_{k}$ as a function of temperature for a three-UC flake (F), a single-UC flake (G), and a half-UC flake (H). Notably, the three-UC flake reveals another FM transition at higher temperatures in addition to the anomaly at the expected bulk $T_{C}$. At the single-UC limit, the bulk transition becomes undetectable, and a single prototypical FM transition is observed at $T_{C}^{\text{ML}}∼35$ K (Fig. 5G), a factor of two larger than the bulk counterpart. At the half-UC limit, we observe a similar transition at $T_{C}^{\text{ML}}$ in the h-BN capped flake, whereas the uncapped flake shows no signal (Fig. 5H). Notably, bulk single crystals also exhibit a small feature at $T_{C}^{\text{ML}}$, as shown in the normalized $\theta_{k}\left(T\right)/\theta_{k}\left(2K\right)$ plot in Fig. 5E, as well as a tiny anomaly in magnetic susceptibility measurements that is consistent with moments from just a couple of β-UTe_3_ layers (see fig. S3b and the Supplementary Materials for details). This result implies that the top and bottom layers of β-UTe_3_ intrinsically display enhanced magnetism with a higher transition temperature compared to the bulk. $T_{C}^{\text{ML}}$ matches the onset of short-range 2D magnetic correlations observed in electrical resistivity, specific heat, and neutron diffraction measurements.

## DISCUSSION

Quantum confinement is generally expected to boost electronic correlations and quantum fluctuations, which act to destabilize a magnetically ordered ground state. The ordering temperature of most vdW magnets at the few-layer limit [e.g., FePS_3_ (*8*, *9*), NiPS_3_ (*51*), MnPSe_3_ (*52*), Cr_2_Ge_2_Te_6_ (*10*), CrI_3_ (*11*), Fe_3_GeTe_2_ (*26*), and CeSiI (*29*)] is either reduced or remains unchanged compared to their bulk counterpart. Our surprising findings point to precisely the opposite: Magnetic order thrives in the 2D limit in β-UTe_3_ flakes and reaches a transition temperature of $T_{C}^{\text{ML}}=35$ K, a factor of two larger than its bulk counterpart.

Given our experimental evidence for 2D magnetic correlations that set in around 30 K in β-UTe_3_, the most likely scenario for our observations is the stabilization of 2D correlations into a higher-temperature ordered state at the surface. Previous theoretical work has established three distinct phase transitions in magnetic systems that display distinct surface ($J_{s}$) and bulk ($J_{b}$) exchange interactions: an “ordinary” transition when $J_{s}\ll J_{b}$, which is the typical transition wherein bulk and surface order at the same temperature; a less common “surface” transition when $J_{s}\gg J_{b}$ and only the surface orders; and a highly uncommon extraordinary transition, which also occurs when $J_{s}\gg J_{b}$ but with bulk order happening at a lower temperature compared to the surface (*53*, *54*). An extraordinary transition requires that the exchange interaction responsible for magnetic ordering is enhanced at the surface compared to the bulk. In 3D magnetic materials, ordinary transitions are commonly observed because their in-plane exchange interactions (J) are comparable to the exchange interactions between neighboring planes ($J_{⊥}$). In this case, $J_{s}$ is therefore expected to be smaller than $J_{b}$ because of the missing $J_{⊥}$ contribution from the top layer. In contrast, in quasi-2D magnetic materials wherein $J\gg J_{⊥}$, small changes on the surface layers may overcome the missing contribution from $J_{⊥}$. As pointed out recently by Guo *et al.* (*55*), quasi-2D systems such as vdW materials are prime candidates for realizing split surface-extraordinary phase transitions, and AFM CrSBr was found to exhibit a 10% increase in ordering temperature in the monolayer limit.

Our results not only support the condition $J_{s}\gg J_{b}$ in β-UTe_3_ but also demonstrate an increase in the ordering temperature by 130% in the monolayer limit. The enhanced magnetic exchange interaction at the surface may be either due to small changes in the exchange interaction due to inversion symmetry breaking at the surface or to a small relaxation of the lattice due to surface strain. Although FM order is enhanced in β-UTe_3_, our thermal expansion measurements along the *b* axis reveal that $T_{C}$ can be quickly suppressed with uniaxial out-of-plane compression with a rapid rate of −91 K/GPa, which provides a powerful avenue for destabilizing magnetism. Our results therefore open numerous avenues for quantum control of electronic correlations, including strain engineering at the monolayer limit. More broadly, our work establishes β-UTe_3_ as a materials platform for investigating, modeling, and controlling the interplay between dimensional confinement, electronic correlations, and magnetism in 2D vdW materials.

## MATERIALS AND METHODS

### Materials synthesis

Single crystals of β-UTe_3_ were prepared using the self-flux technique. U (99.99%) and Te (99.9999%) pieces in a 1:15 ratio were loaded into an alumina crucible and sealed under vacuum in a quartz ampule. The reagents were heated to 875°C, held at 875°C for 100 hours, and slow cooled at 2°C/hour to 550°C. The ampule was then inverted, and the flux was removed via centrifugation. The resultant plate-like crystals were up to 1.2 cm on a side and 1 mm thick (see fig. S9). Scanning electron microscope measurements on an as-grown surface reveal residual Te flux on the surface of the crystals, and energy-dispersive x-ray (EDX) data yield a stoichiometry of UTe_3.8(9)_. EDX measurements repeated on a freshly exfoliated surface yielded a stoichiometry of UTe_3.06(4)_.

### Bulk characterization

Magnetic properties of β-UTe_3_ single crystals were collected on a Quantum Design Magnetic Properties Measurement System with a 7-T magnet and a Quantum Design Physical Property Measurement System (PPMS) with a 16-T and vibrating sample magnetometer options. Magnetic susceptibility and isothermal magnetization measurements were collected with fields parallel and perpendicular to the crystallographic *b* axis.

Specific heat measurements were obtained in PPMS with a ^3^He insert capable of reaching 0.35 K. Measurements to 100 mK were performed in an Oxford Proteox dilution refrigerator. Nonmagnetic analog LaTe_3_ was measured between T=2 to 300 K, and the magnetic entropy of UTe_3_ was obtained by integrating $C_{p}/T$ after subtracting off the lattice contribution of LaTe_3_.

Thermal expansion measurements were performed using a capacitance dilatometer described in (*56*). All thermal expansion measurements were performed using a slow continuous temperature ramp.

Resistivity was measured using an ac resistance bridge. The anisotropic resistivity was determined using the Montgomery method as described in (*57*). For the resistivity derivative, a second sample was measured that was prepared in a bar shape for a standard four-point measurement.

### Exfoliated sample fabrication and measurement

The sample fabrication process involved exfoliating UTe_3_ onto gold-coated silicon substrates (15-nm Au/2-nm Ti/285-nm SiO_2_/Si) immediately after ion polishing the substrate surface using a JEOL IB-19500CP Cross Section Polisher. This method consistently yielded large monolayer and few-layer UTe_3_ flakes (on the order of 100 μm in size), comparable to those obtained by exfoliation onto freshly deposited gold surfaces. In more detail, the substrate was mounted on a rotational stage with its rotation axis near the center of the substrate, and the substrate’s surface aligned parallel to the broad ion beam. Ion polishing was carried out at an accelerating voltage of 4 kV for 1.5 min. Immediately afterward, low-residue Nitto tape carrying freshly cleaved UTe_3_ was applied to the polished substrate. Tape removal was performed inside an argon-filled glovebox with oxygen and water levels below 0.01 parts per million (ppm). Last, selected UTe_3_ flakes were capped with h-BN to further reduce degradation.

To confirm the thickness of the exfoliated flakes, we used an HR-2D AFM system from AFMWorkshop inside the argon-filled glovebox with oxygen and water content less than 0.01 ppm to avoid air degradation. The scans were performed in semicontact mode with a line frequency of 0.5 Hz.

The PKE measurements were performed using a fiber-based, zero-area, Sagnac interferometer with a center frequency of 1550 nm (*58*, *59*). The measurements were performed in a Quantum Design PPMS cryostat with the optical multifunction probe option that provides XYZ sample positioning and a high-resolution camera for imaging the samples. After sample alignment, a shutter can be removed to enable the PKE measurement. The spot size for the PKE measurement was confirmed using a USAF resolution target. For field-dependent measurements, a linear background was subtracted by fitting the data in the fully polarized region. This contribution arises from the effects of the magnetic field on the optical components.

### Neutron diffraction

Neutron diffraction on bulk UTe_3_ was carried out on the time-of-flight diffractometer WISH (STFC, Rutherford Appleton Laboratory) (*60*). WISH is equipped with a solid methane moderator that provides neutron pulses of high brilliance of a broad wavelength bandwidth covering the range between 1 and 10 Å. In combination with the large angular coverage of detector banks, this enables to access a large volume in momentum space.

A UTe_3_ sample of mass (0.26 g) was studied at a fixed orientation at different temperatures. The sample comprises a twin structure associated with a 90° rotation around the vdW axis (010) (see section S10). In addition, the sample displays grain distribution with a mosaicity of the order 3°.

The sample was oriented such that the (010) axis and (001) axis [or (100) axis for the rotated twin] coincided with the horizontal scattering plane. Data were analyzed with the package Mantid (*61*). Diffraction data were described in the reciprocal space of an orthorhombic lattice with lattice parameters a=4.338 Å, b=24.743 Å, and c=4.338 Å. Momentum transfers along (100), (010), and (001) are denoted with a capital Q and characterized in reciprocal lattice units (r.l.u.), which along the three axes are given by $\frac{2\pi }{a}$, $\frac{2\pi }{b}$, and $\frac{2\pi }{c}$, respectively. The diffraction data in the manuscript are presented in momentum space coordinate system $\left(q_{x},q_{y},q_{z}\right)$ with r.l.u. given by $\frac{2\pi }{a}$, $\frac{2\pi }{b}$, and $\frac{2\pi }{a}$. The axis $q_{x}$ corresponds to the vertical direction (referring to the horizontal scattering plane), $q_{y}$ is horizontal and parallel to the K axis common for both twins, and $q_{z}$ is horizontal and parallel to either L or H, depending on the twin that we consider.

Error bars of neutron diffraction data points correspond to uncertainties due to statistical errors. Their size was calculated assuming Poisson counting statistics.

## Supplementary Materials

### This PDF file includes:

Supplementary Text Figs. S1 to S18 Table S1 References
