# Supplementary material for: Enhanced two-dimensional ferromagnetism in van der Waals β-UTe3 monolayers
Source: Sci Adv. 2026 Mar 25;12(13):eaea6436. doi: 10.1126/sciadv.aea6436 (PMC13015880; doi:10.1126/sciadv.aea6436)
Supplement: Supplementary file 1 — Supplementary Text Figs. S1 to S18 Table S1 References [file sciadv.aea6436_sm.pdf]

Science Advances
AAAS

Supplementary Materials for

Enhanced two-dimensional ferromagnetism in van der Waals β-UTe_3 monolayers

Sean M. Thomas *et al.*

Corresponding author: Sean M. Thomas, smthomas@lanl.gov; Priscila F. S. Rosa, pfsrosa@lanl.gov

Sci. Adv. **12**, eaea6436 (2026)
DOI: 10.1126/sciadv.aea6436

This PDF file includes:

Supplementary Text
Figs. S1 to S18
Table S1
References

Supplementary Text

S1. AFM analysis of exfoliated samples

Layer thickness for the polar Kerr effect (PKE) measurements was determined by optical contrast. In order to determine the absolute scale, we compare optical images to atomic force microscopy (AFM) measurements in the same region, as shown in Figure S1. The step height between regions is 1.1–1.2 nm, which corresponds to half of a unit cell along the b axis (2.4743 nm). This is consistent with the $Cmcm$ crystal structure shown in Fig. 1a in the main text, which displays two identical slabs per unit cell separated by a van der Waals gap along the b axis.

S2. Hysteresis in surface ferromagnetism above bulk transition temperature

To confirm the ferromagnetic nature of the enhanced surface magnetism, Kerr rotation was measured as a function of applied field at a temperature above the bulk transition temperature as shown in Fig. S2. Although the magnitude of the Kerr rotation shows thickness dependence, likely due to an optical interaction with the substrate, there is no thickness-dependence of the coercive field. This is somewhat surprising as it suggests there is minimal interaction between the top and bottom surfaces above the bulk transition. This is strikingly different from the behavior below the bulk transition temperature as presented in the main text at 2 K. At low temperatures, β-UTe_3 becomes a softer ferromagnet as the thickness is increased above one unit cell.

S3. Evidence for enhanced surface magnetism in bulk samples

To determine whether the enhanced surface magnetism can be detected in bulk samples, measurements of magnetic susceptibility versus temperature were performed while warming from 1.8 K in zero applied field after cooling in a field of 0.2 T applied along the b axis. As shown in Figure S3, the measured moment at 1.8 K is 2.54 emu/g, or 0.28 μ_B/U. In comparison, a small feature above the background is observed at 29 K, which is about 5 K lower compared with the T_C^{ML} measured on the flakes with 0.5 and 1.0 unit cell thickness. This difference indicates that there may be a slight enhancement of the surface magnetism in the exfoliated flakes due to strain induced by the substrate. The size of the deviation from background is approximately 4×10^{-5} emu/g, or 4.4×10^{-6} μ_B/U. As a result, the moment at 1.8 K is 6×10^4 times larger than the high-temperature anomaly. This

ratio is the same order of magnitude as the number of unit cells along the b axis in a 100 μm-thick sample. This suggests that the feature observed at 29 K is coming from only the top and bottom layers.

S4. Additional specific heat data

Additional specific heat data is shown in Fig. S4. The magnetic contribution to specific heat was determined by subtracting off the value of non-magnetic $LaTe_3$ from the value on β-UTe_3. Fitting C/T versus T^2 yields a Sommerfeld coefficient (γ) of 131 mJ mol^{-1} K^{-2}, which indicates the presence of electronic correlations. In comparison, the value for γ obtained on $LaTe_3$ is less than 1 mJ mol^{-1} K^{-2}.

S5. Additional electrical transport data

Generally, ρ_{zx} can be written as $R_0 H + R_s M$, where the first term is the ordinary Hall component due to the Lorentz force, and R_0 depends on the carrier density. Fig. S5(a) shows ρ_{zx}/H versus M/H at 2 K, which allows for the determination of the ordinary and anomalous contributions. Assuming a single electron band at 2 K would give a carrier concentration of 2.4×10^{23} h^+/cm^3. At intermediate temperatures, ρ_{zx}/H versus M/H acquires curvature. This hinders the determination of the ordinary Hall contribution, especially considering the large ratio between R_s and R_0. At even higher temperatures, M/H collapses to a single value because there is a strictly linear relationship between M and H.

Figures S5(b),(c) show the in-plane magnetoresistance in absolute units and as a percentage, respectively. The magnetoresistance is small and negative above T_C. At 2 K, it remains negative up to the field where the magnetic domains are aligned, then becomes positive in the polarized region.

S6. Thermal expansion

Thermal expansion was measured along the b axis as a function of temperature, as shown in Fig. 3G of the main text. The Ehrenfest relation can be used to determine the pressure dependence of a second-order phase transition through the ratio of the volumetric change ($\Delta\beta V_m$) and the heat

capacity jump ($\Delta C_p/T_C$):

$$\frac{dT_C}{dp} = \frac{\Delta\beta V_m}{\Delta C_p/T_c}. \tag{S1}$$

This is only an approximation for uniaxial pressure, but it is still useful for determining the relative sensitivity of the transition temperature to uniaxial pressure. Using the value of $\Delta C_{\mathrm{mag}}/T_C$ from above (5 mJ mol^{-1} K^{-2}) and the value of $\Delta\alpha_b$ from Fig. 3 (10^{-6} K^{-1}) yields a $\frac{dT_C}{dp}$ of -91 K/GPa. Compared to other measurements, T_C is reduced to about 12 K. This may be due to mounting the crystal in the dilatometer, which induces a small, non-uniform uniaxial pressure along the *b* axis.

S7. Arrott plot

Figure S6 shows an Arrott plot of the magnetization data.

S8. Additional density functional theory (DFT) calculations

Figure S7 shows the DFT+U band structure calculation in the FM state with a Coulomb term $U = 5$ eV.

S9. Bulk single crystal image

Figure S8 shows a photograph of a single crystal of β-UTe_3.

S10. Crystal Structure Refinement

To confirm the crystal structure of UTe_3, which belongs to orthorhombic space group Cmcm, we recorded neutron diffraction data at $T = 25$ K on WISH (ISIS). The data were first formally indexed in the reciprocal space of an orthorhombic lattice with parameters $a = 4.338$ Å, $b = 24.743$ Å, and $c = 4.338$ Å. According to X-ray diffraction, lattice parameters a and c are equal up to the third digit after the comma. Therefore, the definition of axes H and L is not directly clear from the d-spacing of Bragg peaks in time-of flight data and Bragg peaks cannot be directly assigned to the twins in the sample via the d-spacing. For initial analysis steps, we therefore made an arbitrary choice of H and L and indexed Bragg peaks in this coordinate frame.

To integrate Bragg peaks, we utilized the automatic peak detection of the Mantid software. Peaks with ratio of uncertainty σ to integrated intensity I larger than 0.33 were discarded. We

eventually proceeded with a list of 111 integrated Bragg reflections.

In principle, the Bragg peak reflection rules of the Cmcm space group permit to distinguish the two axes H and L from each other. But our data did not allow us to make this decision consistently due to crystal twins that are related by a 90 deg angle around the long van-der-Waals axis. To illustrate this, we performed crystal structure refinements on the integrated data using the software Jana (*62*). We refined an overall scale parameter, a Debye-Waller parameter, an isotropic Gaussian extinction parameter, and the atomic positions. As refinements with anisotropic harmonic Debye-Waller parameters did not improve the refinements, we used isotropic Debye-Waller parameters.

Fig. S9(a) presents the comparison of observed structure factor with the calculated structure factor for the crystal structure model as defined by our initial choice of the axes H and L. The x-axis shows for each Bragg peak the structure factor seen experimentally and the y-axis the structure factor calculated for space group Cmcm in the coordinate frame (HKL) that we initially defined. The large deviation of peaks from $F_{\mathrm{calc}} = F_{\mathrm{obs}}$ (orange line) illustrates the bad agreement of the model. Further, the peaks on the x-axis, which possess odd values of H, are forbidden by the reflection rules of the Cmcm crystal structure and therefore reflect the inadequacy of the refined structural model.

Fig. S9(b) shows the results for alternative definition of momentum space, where the H and L axis are swapped. Although the agreement is much better than before, there are again peaks on the x-axis that possess odd H-coordinate and that are therefore forbidden in the refined structural model.

A considerably better fit is obtained assuming that the sample possesses twins that are related by a 90 deg rotation around the van-der-Waals axis. Fig. S10 shows the results of the refinement, optimizing the scale parameter, the isotropic displacement, extinction, the twin ratio, and the uranium occupancy. The model with twin structure can now account for all peaks that were forbidden for a single-grain structural model and allover the agreement is relatively good. Quantitatively, the refinement is characterized by the goodness of fit parameters $GoF = 7.68$, $R = 8.88$, and $wR2 = 19.98$. The twin ratio determined in the refinement indicates that 13 percent are in the (HKL)-domain, see S9(a), and 87 percent in the (LKH) domain, see (b). The uranium occupancy is given by 92(1) percent. Isotropic displacement and extinction parameters refined to $G_{\mathrm{iso}} = 0.015(2)$ and $G_{\mathrm{iso}} = 0.0030(8)$.

S11. Magnetic Structure Determination

To determine the ordered magnetic moment of UTe_3, we performed neutron diffraction at T = 1.5 K. Magnetic Bragg peaks were observed at the same momentum transfers as structural Bragg peaks, therefore suggesting that the scattering is located at the Γ-point associated with wave-vector $\boldsymbol{k}_0 = (0, 0, 0)$.

In total, 108 Bragg reflections were indexed and integrated (as before, peaks with low statistics were discarded). The refinement of magnetic structures with wave-vector $(0, 0, 0)$ is frequently challenging, as magnetic intensities coincide with nuclear Bragg intensities in momentum space. In addition, magnetic symmetry analysis may provide many different structures that display almost the same diffraction pattern. Group theoretical magnetic symmetry analysis reveals six representations for the wave-vector $\boldsymbol{k}_0 = (0, 0, 0)$, which are denoted mGM2+, mGM3+, mGM4+, mGM1-, mGM2-, and mGM3-. The first three representations are ferro- and the last three antiferromagnetic.

As bulk measurements on UTe_3 suggested ferromagnetism with magnetic moments along the b-axis and, in addition, signatures of antiferromagnetic components are absent in our diffraction data, we probed three different magnetic structures, namely moments along the c-axis (mGM2+), along the a-axis (mGM3+), and along the b-axis (mGM4+). For the refinements, we fixed the allover scale parameter, the extinction parameter and the twin fraction to the value inferred from structural refinements and fitted the the isotropic displacement parameter as well as all components of magnetic moments that are permitted in the respective irreducible representation.

We further assumed magnetism carried by magnetic U^{3+} ions with formfactor:

$$f = \langle j_0 \rangle + c_2 \cdot \langle j_2 \rangle \ . \tag{S2}$$

For the radial integrals of spherical Bessel functions, $\langle j_n \rangle$, we took the values tabulated in Ref. (*63*). For the constant, c_2, we considered the value obtained for the Hunds-rule Russell-Saunders ground state given by $c_2 = 1.75$ (see also Ref. (*63*)).

Fig. S11 compares the structure factors of the three refined models with the neutron diffraction data. A summary of all fit-parameters is further provided in Tab. S1. The plots and the goodness of fit parameters (GoF) look relatively similar and the refinements themselves do not permit to distinguish the magnetic structures. However, as we explain below, the structure factor on well selected momentum space positions clearly shows that magnetic moments are essentially aligned

along the b-axis with a small possible canting angle.

The refined magnetic moment obtained for the best fit with mGM4+ is given by $M = 0.48(11)\,\mu_B$.

In order to further improve the refinement results, we superposed the irreducible representation mGM4+ with other irreducible representations associated with $\boldsymbol{k} = (0,0,0)$ and $\boldsymbol{k} = (1,0,0)$. But none of these superpositions of two IRs resulted in a converging refinement with better statistics. Taken together, refinements therefore suggest magnetic long-range order with wave-vector $\boldsymbol{k} = (0,0,0)$ and magnetic moments aligned along the b-axis.

Neutron diffraction data at carefully selected momentum-space positions show further that the magnetic ground state has almost negligible projection perpendicular to the b-axis. Therefore, the ground state corresponds essentially to ferromagnetic order with moments aligned along the b-axis.

Fig. S12 shows temperature subtracted diffraction data on the line $(0, K, 0)$. The peak at $K = 2$ and the absence of intensity at $K = 1$ indicate the presence of a weak $\boldsymbol{k} = 0$ component perpendicular to the b-axis as well as the absence of an antiferromagnetic component.

To assess the magnitude of components perpendicular to b (denoted $m_\perp$) and along b (denoted $m_{||}$), we consider integrated intensities of specific deliberately chosen magnetic Bragg peaks. The magnetic Bragg intensity at (020) is purely due to components perpendicular to b, denoted $m_\perp$, and given by $I_m(020) = 2454 \pm 1036$. The Bragg peak at (130), in turn, is due to both $m_\perp$ and $m_{||}$ and has intensity $I_m(130) = 194333 \pm 5448$.

Comparing these intensities with the magnetic structure factor calculated for collinear ferromagnetic spin texture with moment projections both along $m_\perp$ and $m_{||}$ (with U^{3+} form factor and neglected Debye-Waller factor) yields a ratio $m_\perp = m_{||} \cdot 0.07(4)$, where $m_\perp$ points either along [100] or [001].

Taken together, the comparison of integrated intensities at (130) and (020) show that the ground state is essentially a b-axis ferromagnet, possibly with a tiny tilt of moments either along [100] or [001].

S12. Mosaicity and integration of Bragg peaks

Our sample used in neutron diffractions showed two separate crystals misaligned by a couple of degrees each showing 90 degrees twinning. Fig. S13 illustrates this showing an exemplary detector

image taken on WISH. Peaks clearly display splitting (bright spots). The grains are spread over an angular range of the order 3 deg. For example the two red dots are separated by a horizontal angle $\Delta\phi = 2.1$ deg and a vertical angle $\Delta\Psi = 0.7$ deg.

For integration, we chose radii on the detector large enough to cover Bragg peak of all grains around a chosen position.

S13. Magnetic wave-vector inferred from neutron scattering

To determine magnetic ordering vectors at low temperature, we performed neutron diffraction below the transition temperature (at $T = 1.5$ K) and above the transition temperature (at $T = 25$ K). Data are presented in Fig. S14.

Fig. S15 compares the two diffraction data-sets. Magnetic scattering intensity is observed at integer-valued momentum transfers (h, k, l), therefore suggesting magnetic ordering wave-vector $\boldsymbol{k} = (0, 0, 0)$ (Γ-point). Magnetic ordering at the Y-point would be antiferromagnetic and would result in diffraction intensity at momentum-transfers, where structural Bragg peaks are absent, such as (030). In our data-set, we only observed magnetic scattering at the same momentum-transfers, where structural Bragg peaks appear, whereas $(0, 3, 0)$ do not display any magnetic scattering, therefore suggesting that the magnetic ordering wave-vector is $\boldsymbol{k} = (0, 0, 0)$ (Γ-point). As bulk characterization suggested ferromagnetism, we conclude, that the wave-vector is indeed at the Γ-point.

Magnetic Bragg peaks display the same width in all momentum space-directions as structural Bragg peaks, indicating the three-dimensional nature of the magnetic long-range order. Fig. S16 illustrates this, showing cuts through $\boldsymbol{Q} = (0, -3, 1)$ along the three different momentum-space directions K, L, and H.

S14. Transition temperature inferred from neutron scattering

The temperature dependence of magnetic order parameter was inferred from the magnetic Bragg peak at (310), where magnetic scattering intensity was strongest.

The thermal variation of the magnetic intensity is shown in Fig. 2**b** of the main text and was

fitted with order parameter curves:

$$I(T) := I_0 \cdot \left(1 - \frac{T}{Tc}\right)^{2\beta} \tag{S3}$$

by means of least-squares fits.

In order to determine the transition temperature T_c, we repeated the fit for different values of T_c and made an assessment of the fit based on the χ^2 values calculated on all data-points below the respective T_c. Fig. S17 shows the goodness of the fit as a function of T_c. The best fit (lowest χ^2) is obtained for $T_c = 15.9$ K. We further find that χ^2 is smaller than 1 for temperatures $15.3 \leq T_c \leq 16.8$ and take the respective temperature range as uncertainty for T_c. Taken together, the transition temperature is given by $T_c = 15.9(9)$ K

S15. Correlation length of diffuse magnetic scattering

In order to obtain an estimate of the correlation length of the diffuse magnetic scattering observed along the line $\boldsymbol{Q} = (0, K, 0)$, we integrated the diffuse scattering presented in Fig. 2**c** of the main text along the temperature-axis. The resulting intensity as a function of ΔL is presented in Fig. S18.

The profile displays a Gaussian shape with full width at half maximum $f_1 = 1.79 \cdot 10^{-1}$ r.l.u.. The peak profile is a convolution of intrinsic peak-width, f_0, and experimental resolution, f_R. Assuming a Gaussian profile for resolution and intrinsic peak width, we obtain $f_1 = \sqrt{f_0^2 + f_R^2}$.

The experimental resolution can be inferred from the peak at $\boldsymbol{Q} = (0, 2, 0)$, which along the L-axis exhibits a full width at half maximum given by $f_R = 6.9 \cdot 10^{-3}$ r.l.u.. Intrinsic peak-width is therefore given by $f_0 = 1.70 \cdot 10^{-1}$ r.l.u. and the correlation length along the L-axis is given by $\kappa_0 = 2/f_0 = 7.7$ Å.

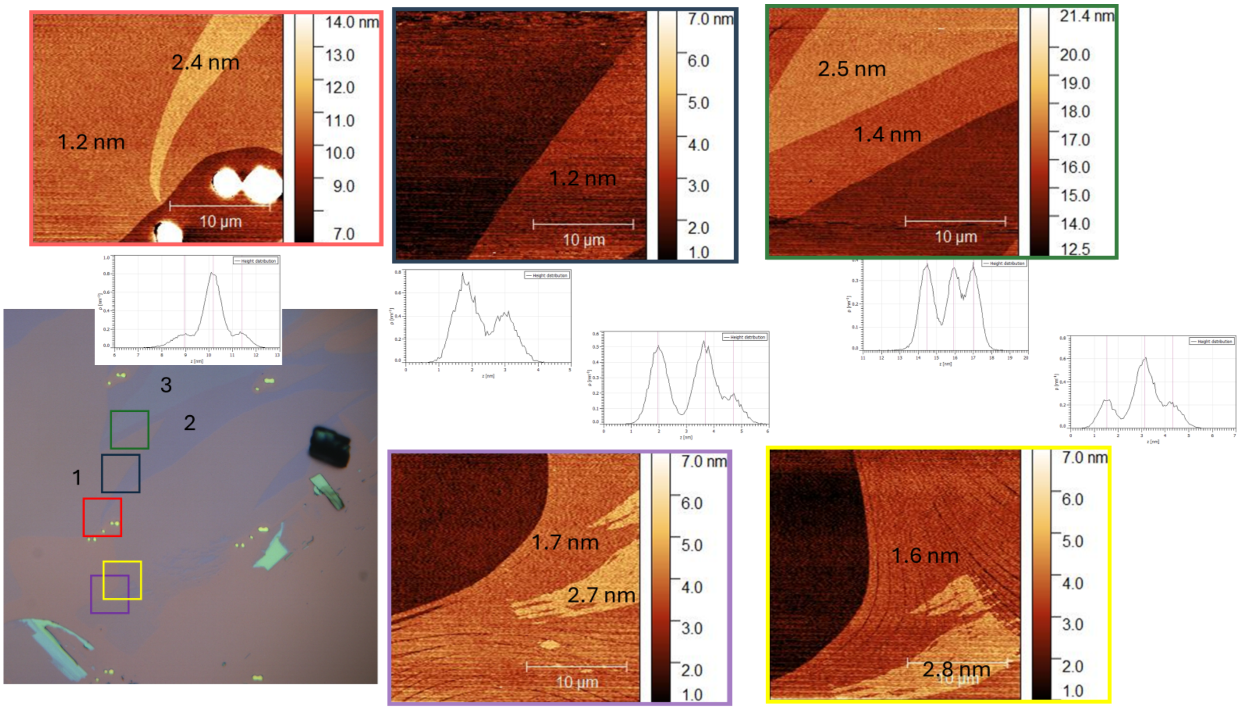

Figure S1: Bottom-right shows an optical image of exfoliated UTe_3 along with colored squares showing the field-of-view for matching AFM measurements. The colored squares correspond with the border color of the height maps shown in the remainder of the panel.

Table S1: Magnetic structure refinements. The table summarizes the values R, $wR2$, and GoF for the refinement of the three models mGM2+, mGM3+, and mGM4+ with data recorded at $T = 1.5$ K. Values R and $wR2$ are shown separately for magnetic and nuclear Bragg peaks. Although mGM3+ and mGM4+ GoF are nearly identical, consideration of the (130) and (020) peak intensities identify mGM4+ as the correct magnetic order (see SI text).

IR	R_{struc}	$wR2_{struc}$	R_{mag}	$wR2_{mag}$	GoF
mGM2+	12.08	24.25	21.23	39.42	11.02
mGM3+	10.44	20.75	19.77	38.21	10.36
mGM4+	10.64	22.74	20.05	38.50	10.51

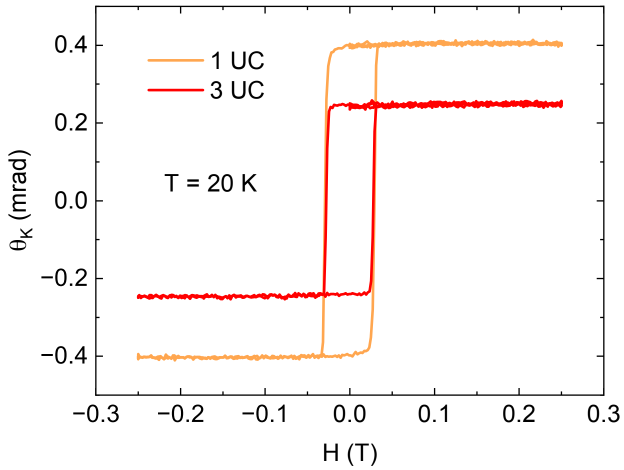

Figure S2: Kerr rotation versus field at 20 K for two different thicknesses. A linear background (due to the effect of the magnetic field on the optics) was subtracted by fitting the data in the fully-polarized region (H > 0.1 T). The coercivity is the same for both thickness (±28 mT).

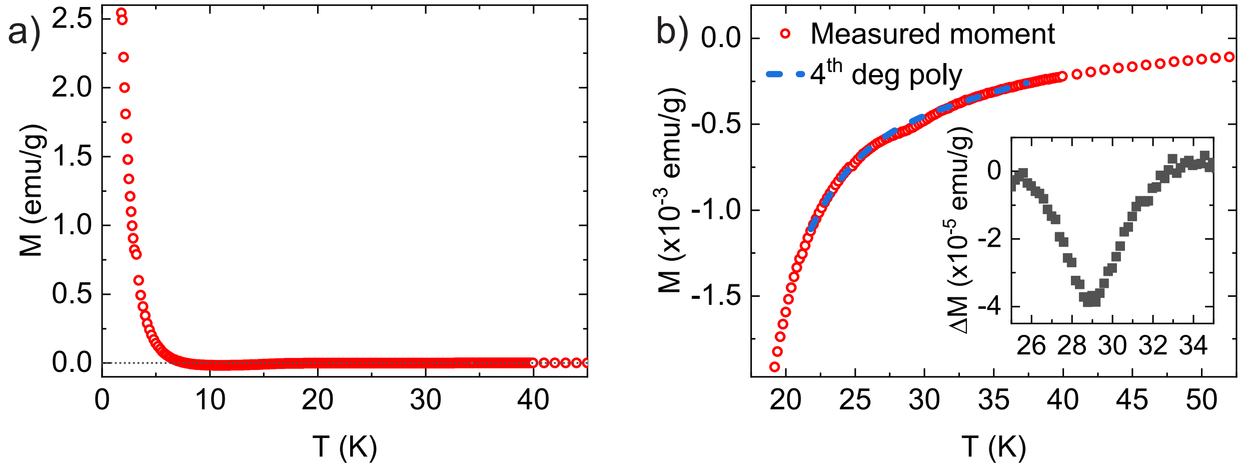

Figure S3: Magnetic moment versus temperature while warming in zero field after cooling in a 0.2 T field applied along the *b* axis. (a) A zoomed-out view showing a moment of about 2.5 emu/g at 1.8 K. (b) A zoomed-in view showing the small anomaly measured near 29 K. The inset shows the difference in the moment between a background polynomial fit and the measured data.

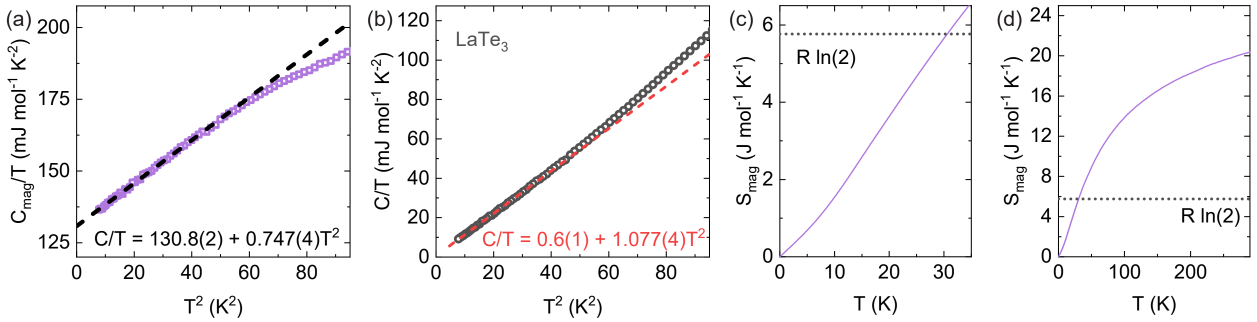

Figure S4: (a) Magnetic specific heat ($C_{\beta UTe_3}-C_{LaTe_3}$) divided by temperature versus temperature squared. (b) Specific heat of $LaTe_3$ divided by temperature versus temperature squared. (c) Integrated magnetic entropy versus temperature. The dashed line indicates the value Rln 2. This is the expected entropy recovered for a ground-state doublet. (d) Same as (c), but over a wider temperature range.

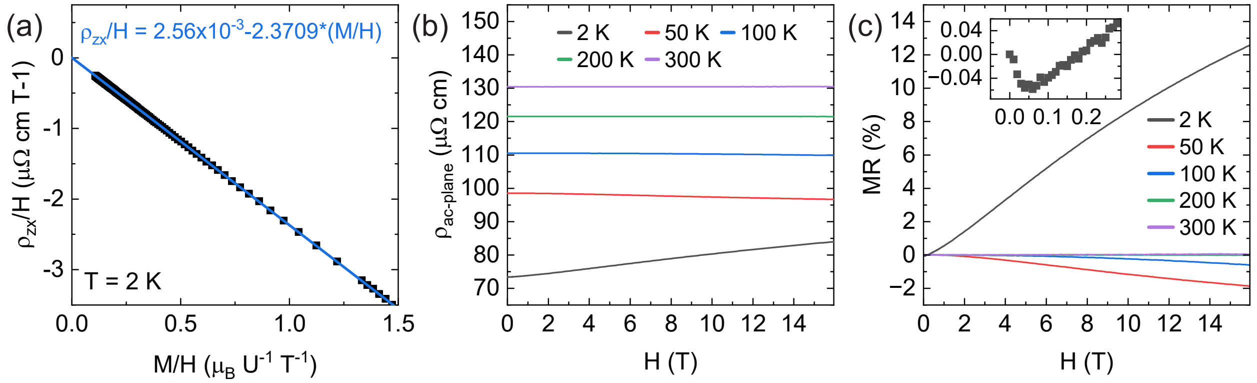

Figure S5: (a) ρ_{zx}/H versus M/H at 2 K. (b) In-plane magnetoresistance versus field. (c) Magnetoresistance percentage $[\rho(H)-\rho(0)]/\rho(0)$ versus field. The inset shows a zoomed-in view for the 2 K data at low field. The magnetic field is applied parallel to the b axis for all plots.

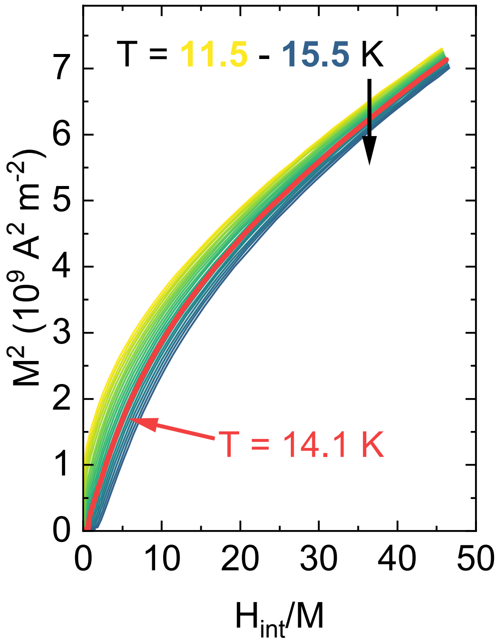

Figure S6: An Arrott plot for temperatures near T_C indicates that the transition temperature is approximately 14.1 K (red curve).

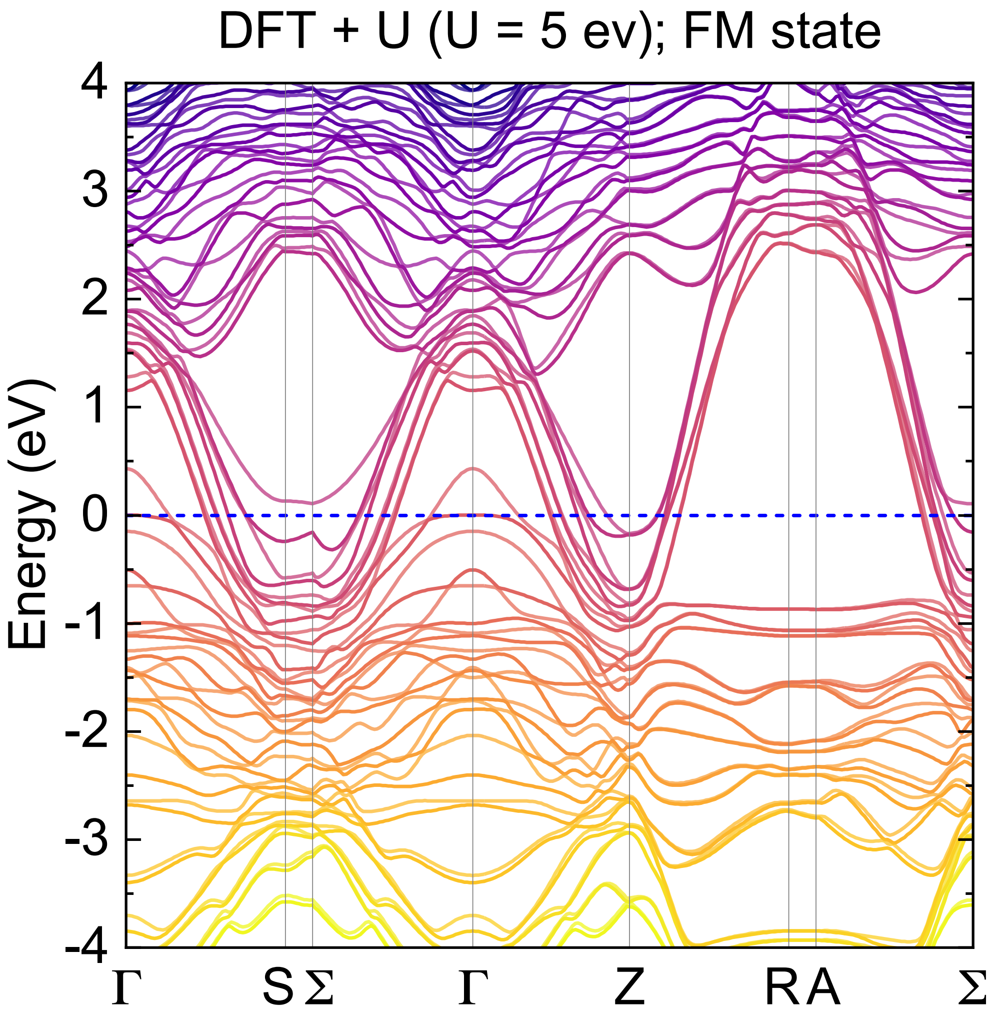

Figure S7: DFT+U band structure calculation in the FM state with a Coulomb term $U = 5$ eV.

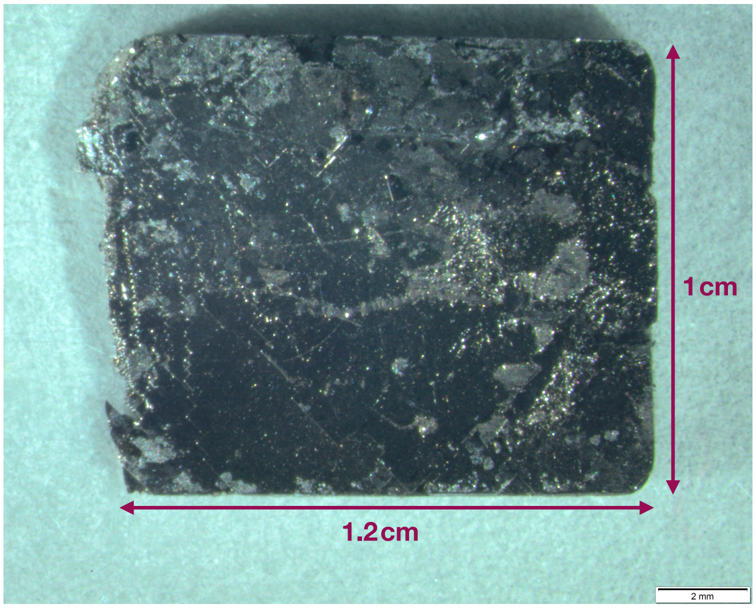

Figure S8: Image of a large bulk single crystal of β-UTe_3.

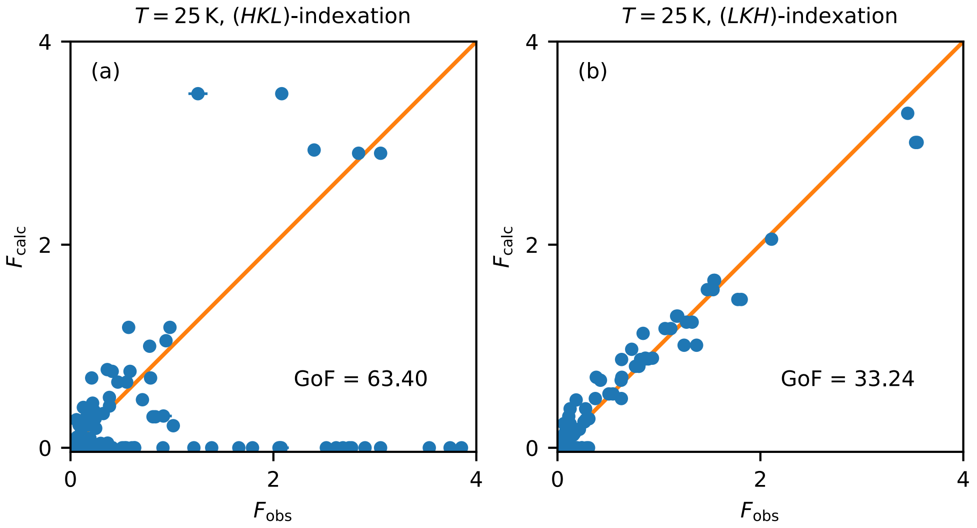

Figure S9: Crystal structure refinement of a single-grain Cmcm structure. Experimentally observed structure factor inferred from Bragg peaks, F_{obs}, is compared with the calculated structure factor of a Cmcm structure with (a) momentum-space coordinates (HKL) and (b) with coordinates (LKH). The orange line denotes the region, where $F_{\text{calc}} = F_{\text{obs}}$.

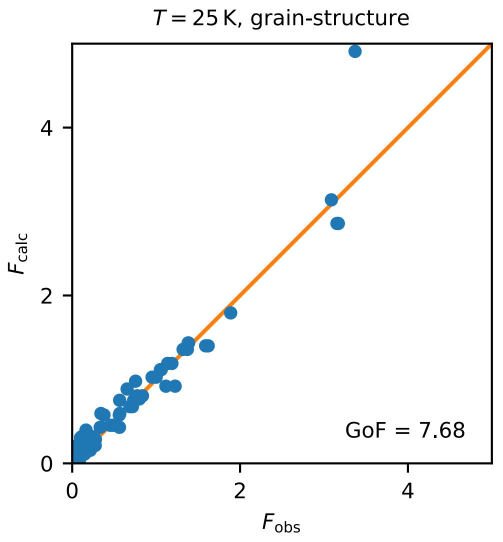

Figure S10: Calculated vs. observed structure factor for a crystal structure refinement with twin structure. The twins are related by a 90 deg rotation around the vdW axis.

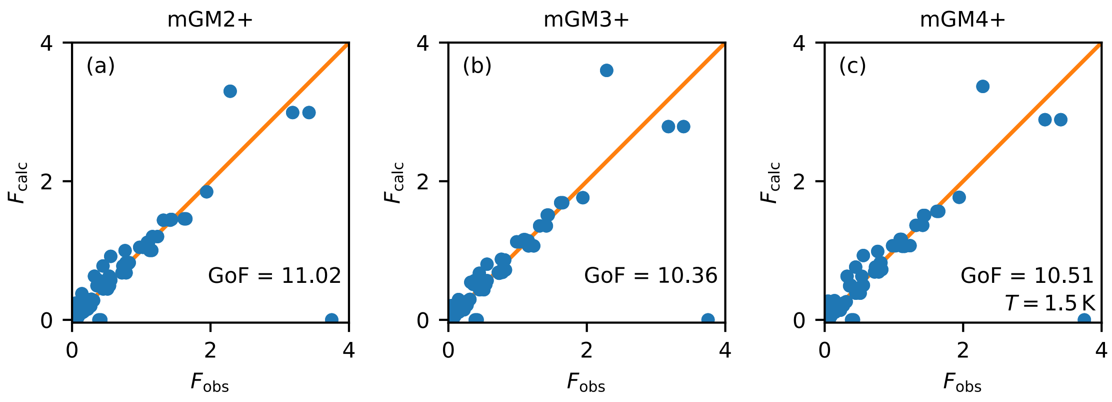

Figure S11: Combined magnetic and crystal structure refinements at $T = 1.5$ K. Neutron diffraction data, comprising of a list of 108 integrated Bragg intensities, were compared to ferromagnetic spin textures with moments (a) along the c-axis, (b) along the a-axis, and (c) along the b-axis. The blue data points compare for each Bragg peak the recorded and calculated structure factor.

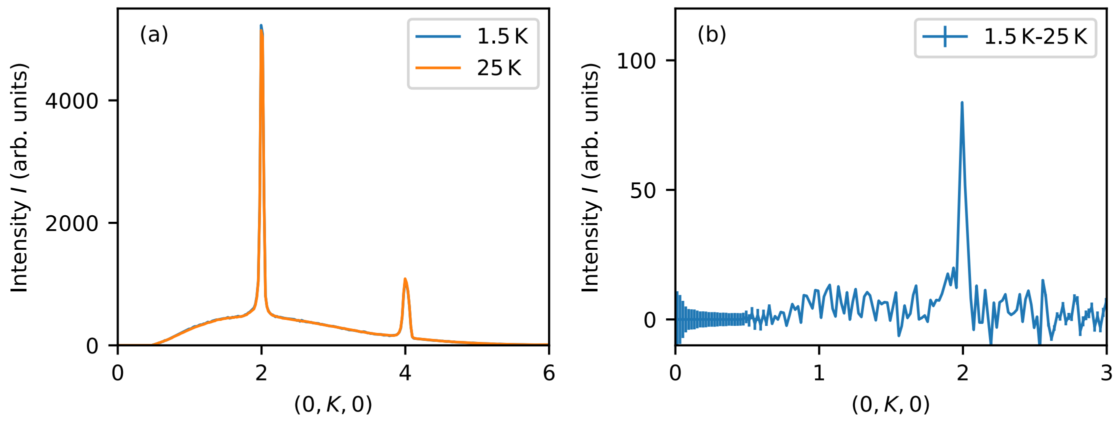

Figure S12: Magnetic ordering vector at $T = 1.5$ K. Neutron diffraction data shown were recorded at $T = 1.5$ K and at $T = 25$ K. The thermal variation displays Bragg intensity at the same momentum transfers as nuclear Bragg diffraction, therefore suggesting zero momentum wave-vector.

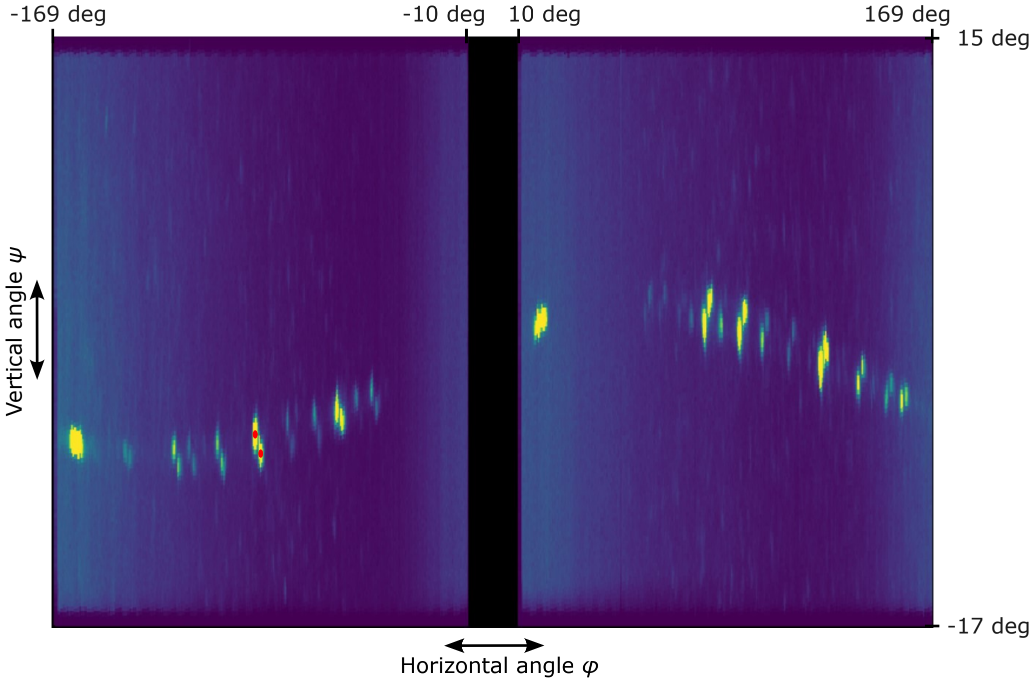

Figure S13: Typical detector image recorded on Wish. The exemplary image was taken at $T = 1.5$ K. The bright spots are associated with Bragg peaks. The splitting of Bragg peaks is due to finite mosaicity and grains in our sample. The two red dots correspond to the same Bragg peaks in different grains.

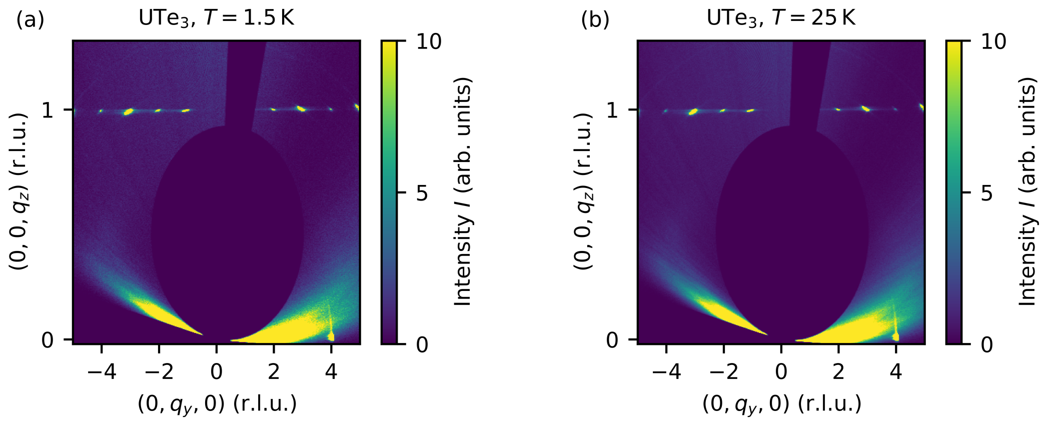

Figure S14: Magnetic ordering vector at $T = 1.5$ K. Neutron diffraction data shown on the lines (a) $(0, K, 1)$ and (a) $(0, K, 2)$ were recorded at $T = 1.5$ K and at $T = 25$ K. The thermal variation displays Bragg intensity at the same momentum transfers as nuclear Bragg diffraction, therefore suggesting wave-vector at zero momentum.

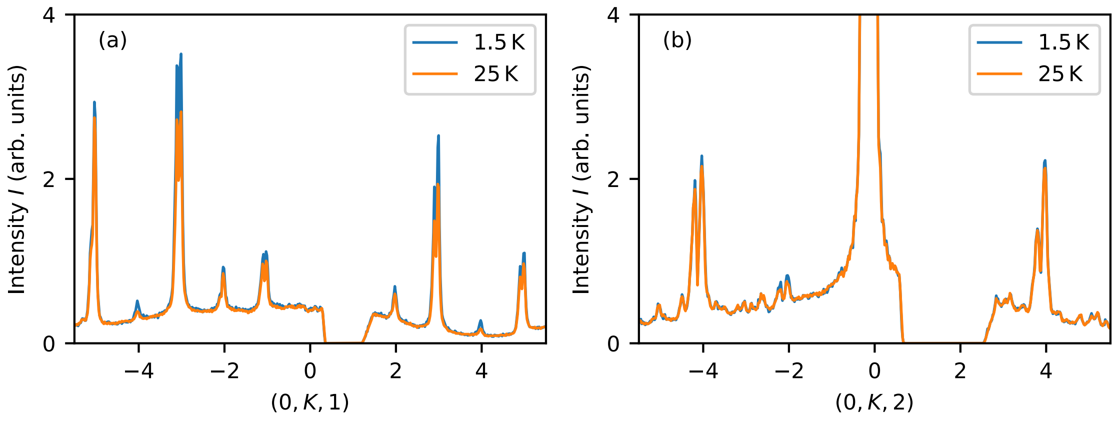

Figure S15: Magnetic ordering vector at $T = 1.5$ K. Neutron diffraction data shown on the lines (a) $(0, K, 1)$ and (a) $(0, K, 2)$ were recorded at $T = 1.5$ K and at $T = 25$ K. The thermal variation displays Bragg intensity at the same momentum transfers as nuclear Bragg diffraction, therefore suggesting wave-vector at zero momentum.

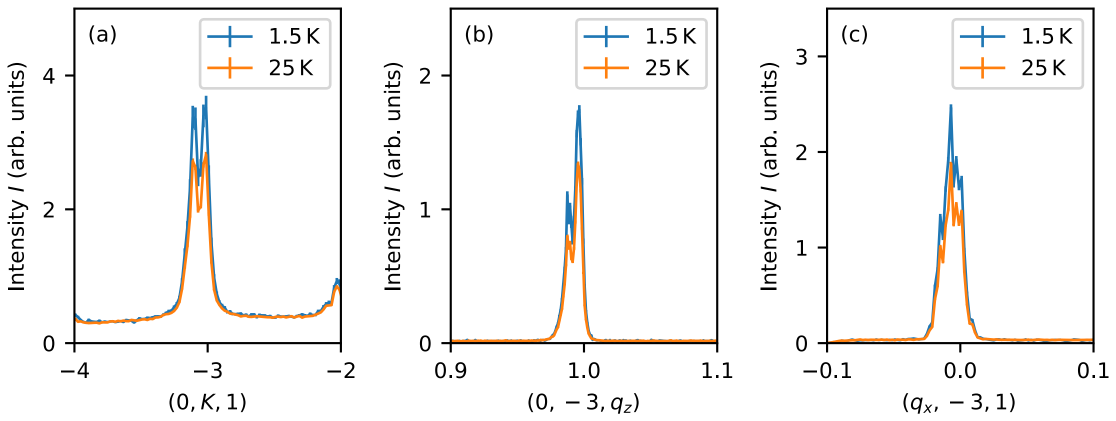

Figure S16: Cut through neutron diffraction data around the Bragg peak at $(0, -3, 1)$. Along all three momentum-space directions (a) H, (b) q_z, and (c) q_x, the magnetic Bragg peak displays the same width as the structural Bragg peak.

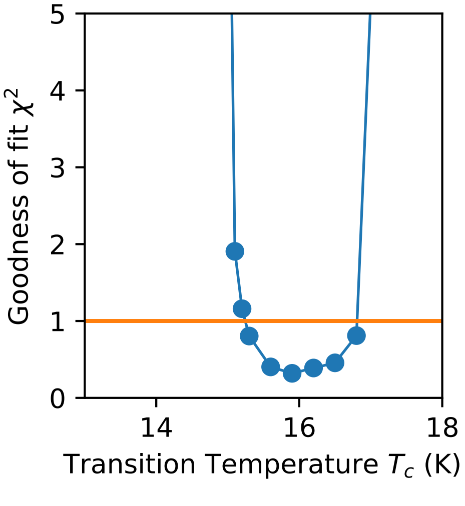

Figure S17: Goodness of order-parameter fit to neutron diffraction data. The circle symbols denote the χ^2 values obtained for different transition temperatures. The orange line corresponds to the border of $\chi^2 \leq 1$, which we considered as acceptable range for the order-parameter fit.

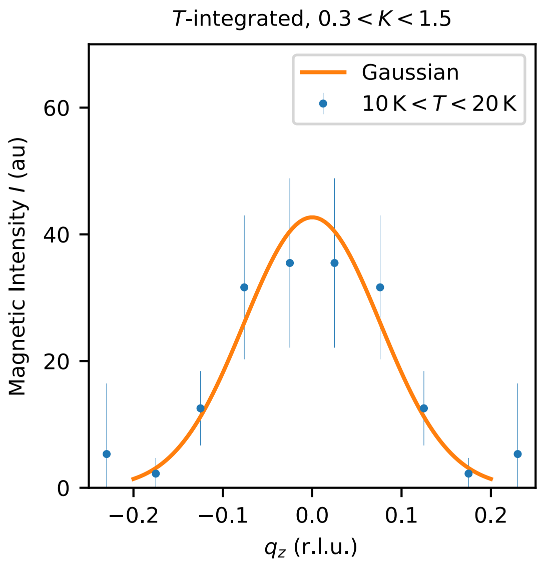

Figure S18: Diffuse magnetic scattering intensity integrated over the temperature range from 10 K to 20 K for different values of q_z. The orange line corresponds to a Gaussian peak shape with FWHM $f_1 = 3.6 \cdot 10^{-2}$ r.l.u..

REFERENCES

1. K. S. Novoselov, A. K. Geim, S. V. Morozov, D. Jiang, Y. Zhang, S. V. Dubonos, I. V. Grigorieva, A. A. Firsov, Electric field effect in atomically thin carbon films. *Science* **306**, 666–669 (2004).

2. E. Y. Andrei, A. H. MacDonald, Graphene bilayers with a twist. *Nat. Mater.* **19**, 1265–1275 (2020).

3. Y. Xie, A. T. Pierce, J. M. Park, D. E. Parker, E. Khalaf, P. Ledwith, Y. Cao, S. H. Lee, S. Chen, P. R. Forrester, K. Watanabe, T. Taniguchi, A. Vishwanath, P. Jarillo-Herrero, A. Yacoby, Fractional Chern insulators in magic-angle twisted bilayer graphene. *Nature* **600**, 439–443 (2021).

4. T. Han, Z. Lu, Z. Hadjri, L. Shi, Z. Wu, W. Xu, Y. Yao, A. A. Cotten, O. Sharifi Sedeh, H. Weldeyesus, J. Yang, J. Seo, S. Ye, M. Zhou, H. Liu, G. Shi, Z. Hua, K. Watanabe, T. Taniguchi, P. Xiong, D. M. Zumbühl, L. Fu, L. Ju, Signatures of chiral superconductivity in rhombohedral graphene. *Nature* (2025), **643**, 654, 661.

5. K. S. Novoselov, A. Mishchenko, A. Carvalho, A. H. C. Neto, 2D materials and van der Waals heterostructures. *Science* **353**, aac9439 (2016).

6. C. Tan, X. Cao, X. J. Wu, Q. He, J. Yang, X. Zhang, J. Chen, W. Zhao, S. Han, G.-H. Nam, M. Sindoro, H. Zhang, Recent advances in ultrathin two-dimensional nanomaterials. *Chem. Rev.* **117**, 6225–6331 (2017).

7. A. Castellanos-Gomez, X. Duan, Z. Fei, H. R. Gutierrez, Y. Huang, X. Huang, J. Quereda, Q. Qian, E. Sutter, P. Sutter, Van der Waals heterostructures. *Nat. Rev. Methods Primers* **2**, 58 (2022).

8. J.-U. Lee, S. Lee, J. H. Ryoo, S. Kang, T. Y. Kim, P. Kim, C.-H. Park, J.-G. Park, H. Cheong, Ising-type magnetic ordering in atomically thin ($FePS_3$). *Nano Lett.* **16**, 7433–7438 (2016).

9. X. Wang, K. du, Y. Y. Fredrik Liu, P. Hu, J. Zhang, Q. Zhang, M. H. S. Owen, X. Lu, C. K. Gan, P. Sengupta, C. Kloc, Q. Xiong, Raman spectroscopy of atomically thin two-dimensional magnetic iron phosphorus trisulfide (FePS3) crystals. *2D Materials* **3**, 031009 (2016).

10. C. Gong, L. Li, Z. Li, H. Ji, A. Stern, Y. Xia, T. Cao, W. Bao, C. Wang, Y. Wang, Z. Q. Qiu, R. J. Cava, S. G. Louie, J. Xia, X. Zhang, Discovery of intrinsic ferromagnetism in two-dimensional van der Waals crystals. *Nature* **546**, 265–269 (2017).

11. B. Huang, G. Clark, E. Navarro-Moratalla, D. R. Klein, R. Cheng, K. L. Seyler, D. Zhong, E. Schmidgall, M. A. McGuire, D. H. Cobden, W. Yao, D. Xiao, P. Jarillo-Herrero, X. Xu, Layer-dependent ferromagnetism in a van der Waals crystal down to the monolayer limit. *Nature* **546**, 270–273 (2017).

12. K. S. Burch, D. Mandrus, J.-G. Park, Magnetism in two-dimensional van der Waals materials. *Nature* **563**, 47–52 (2018).

13. Q. H. Wang, A. Bedoya-Pinto, M. Blei, A. H. Dismukes, A. Hamo, S. Jenkins, M. Koperski, Y. Liu, Q. C. Sun, E. J. Telford, H. H. Kim, M. Augustin, U. Vool, J.-X. Yin, L. H. Li, A. Falin, C. R. Dean, F. Casanova, R. F. L. Evans, M. Chshiev, A. Mishchenko, C. Petrovic, R. He, L. Zhao, A. W. Tsen, B. D. Gerardot, M. Brotons-Gisbert, Z. Guguchia, X. Roy, S. Tongay, Z. Wang, M. Z. Hasan, J. Wrachtrup, A. Yacoby, A. Fert, S. Parkin, K. S. Novoselov, P. Dai, L. Balicas, E. J. G. Santos, The magnetic genome of two-dimensional van der Waals materials. *ACS Nano* **16**, 6960–7079 (2022).

14. M. Gibertini, M. Koperski, A. F. Morpurgo, K. S. Novoselov, Magnetic 2D materials and heterostructures. *Nat. Nanotechnol.* **14**, 408–419 (2019).

15. M. E. Ziebel, M. L. Feuer, J. Cox, X. Zhu, C. R. Dean, X. Roy, CrSBr: An air-stable, two-dimensional magnetic semiconductor. *Nano Lett.* **24**, 4319–4329 (2024).

16. C. M. Varma, Quantum-critical fluctuations in 2D metals: Strange metals and superconductivity in antiferromagnets and in cuprates. *Rep. Prog. Phys.* **79**, 082501 (2016).

17. P. W. Phillips, N. E. Hussey, P. Abbamonte, Stranger than metals. *Science* **377**, 6602 (2022).

18. J. Alicea, New directions in the pursuit of Majorana fermions in solid state systems. *Rep. Prog. Phys.* **75**, 076501 (2012).

19. C. Broyles, S. Mardanya, M. Liu, J. Ahn, T. Dinh, G. Alqasseri, J. Garner, Z. Rehfuss, K. Guo, J. Zhu, D. Martinez, D. Li, Y. Hao, H. Cao, M. Boswell, W. Xie, J. G. Philbrick, T. Kong, L. Yang, A. Vishwanath, P. Kim, S.-Y. Xu, J. E. Hoffman, J. D. Denlinger, S. Chowdhury, S. Ran, UOTe: Kondo-interacting topological antiferromagnet in a Van der Waals lattice. *Adv. Mater.* **37**, 2414966 (2025).

20. K. Yumigeta, Y. Qin, H. Li, M. Blei, Y. Attarde, C. Kopas, S. Tongay, Advances in rare-earth tritelluride quantum materials: Structure, properties, and synthesis. *Adv. Sci.* **8**, e2004762 (2021).

21. N. Ru, I. R. Fisher, Thermodynamic and transport properties of YTe_3, $LaTe_3$, and $CeTe_3$. *Phys. Rev. B* **73**, 033101 (2006).

22. S. Lei, J. Lin, Y. Jia, M. Gray, A. Topp, G. Farahi, S. Klemenz, T. Gao, F. Rodolakis, J. L. McChesney, C. R. Ast, A. Yazdani, K. S. Burch, S. Wu, N. P. Ong, L. M. Schoop, High mobility in a van der Waals layered antiferromagnetic metal. *Sci. Adv.* **6**, eaay6407 (2020).

23. J.-X. Zhu, M. Janoschek, D. S. Chaves, J. C. Cezar, T. Durakiewicz, F. Ronning, Y. Sassa, M. Mansson, B. L. Scott, N. Wakeham, E. D. Bauer, J. D. Thompson, Electronic correlation and magnetism in the ferromagnetic metal Fe_3GeTe_2. *Phys. Rev. B* **93**, 144404 (2016).

24. A. F. May, D. Ovchinnikov, Q. Zheng, R. Hermann, S. Calder, B. Huang, Z. Fei, Y. Liu, X. Xu, M. A. McGuire, Ferromagnetism near room temperature in the cleavable van der Waals crystal Fe_5GeTe_2. *ACS Nano* **13**, 4436–4442 (2019).

25. Z. Fei, B. Huang, P. Malinowski, W. Wang, T. Song, J. Sanchez, W. Yao, D. Xiao, X. Zhu, A. F. May, W. Wu, D. H. Cobden, J.-H. Chu, X. Xu, Two-dimensional itinerant ferromagnetism in atomically thin Fe_3GeTe_2. *Nat. Mater.* **17**, 778–782 (2018).

26. Y. Deng, Y. Yu, Y. Song, J. Zhang, N. Z. Wang, Z. Sun, Y. Yi, Y. Z. Wu, S. Wu, J. Zhu, J. Wang, X. H. Chen, Y. Zhang, Gate-tunable room-temperature ferromagnetism in two-dimensional Fe_3GeTe_2. *Nature* **563**, 94–99 (2018).

27. H. Wang, H. Lu, Z. Guo, A. Li, P. Wu, J. Li, W. Xie, Z. Sun, P. Li, H. Damas, A. M. Friedel, S. Migot, J. Ghanbaja, L. Moreau, Y. Fagot-Revurat, S. Petit-Watelot, T. Hauet, J. Robertson, S. Mangin, W. Zhao, T. Nie, Interfacial engineering of ferromagnetism in wafer-scale van der Waals Fe_4GeTe_2 far above room temperature. *Nat. Commun.* **14**, 2483 (2023).

28. N.-T. Dang, D. P. Kozlenko, O. N. Lis, S. E. Kichanov, Y. V. Lukin, N. O. Golosova, B. N. Savenko, D.-L. Duong, T.-L. Phan, T.-A. Tran, M.-H. Phan, High pressure-driven magnetic disorder and structural transformation in Fe3GeTe2: Emergence of a magnetic quantum critical point. *Adv. Sci.* **10**, e2206842 (2023).

29. V. A. Posey, S. Turkel, M. Rezaee, A. Devarakonda, A. K. Kundu, C. S. Ong, M. Thinel, D. G. Chica, R. A. Vitalone, R. Jing, S. Xu, D. R. Needell, E. Meirzadeh, M. L. Feuer, A. Jindal, X. Cui, T. Valla, P. Thunström, T. Yilmaz, E. Vescovo, D. Graf, X. Zhu, A. Scheie, A. F. May, O. Eriksson, D. N. Basov, C. R. Dean, A. Rubio, P. Kim, M. E. Ziebel, A. J. Millis, A. N. Pasupathy, X. Roy, Two-dimensional heavy fermions in the van der Waals metal CeSiI. *Nature* **625**, 483–488 (2024).

30. C. J. Peters, R. J. Birgeneau, M. A. Kastner, H. Yoshizawa, Y. Endoh, J. Tranquada, G. Shirane, Y. Hidaka, M. Oda, M. Suzuki, T. Murakami, Two-dimensional zone-center spin-wave excitations in La_2CuO_4. *Phys. Rev. B* **37**, 9761–9764 (1988).

31. K. Sun, J. H. Cho, F. C. Chou, W. C. Lee, L. L. Miller, D. C. Johnston, Y. Hidaka, T. Murakami, Heat capacity of single-crystal La_2CuO_4 and polycrystalline $La_{2-x}Sr_xCuO_4$ ($0 \leq x \leq 0.20$) from 110 to 600 K. *Phys. Rev. B* **43**, 239 (1991), 246.

32. G. R. Stewart, Heavy-fermion systems. *Rev. Mod. Phys.* **56**, 755–787 (1984).

33. B. White, J. Thompson, M. Maple, Unconventional superconductivity in heavy-fermion compounds. *Phys. C, Supercond. Appl.* **514**, 246–278 (2015).

34. D. Aoki, K. Ishida, J. Flouquet, Review of U-based ferromagnetic superconductors: Comparison between UGe_2, URhGe, and UCoGe. *J. Phys. Soc. Jpn.* **88**, 022001 (2019).

35. D. Aoki, J.-P. Brison, J. Flouquet, K. Ishida, G. Knebel, Y. Tokunaga, Y. Yanase, Unconventional superconductivity in UTe_2. *J. Phys. Condens. Matter* **34**, 243002 (2022).

36. H. Noel, J. Levet, Caractérisation d'un tritellurure d'uranium: β-UTe_3 de structure type $NdTe_3$. *J. Solid State Chem.* **79**, 28–33 (1989).

37. M. Sundermann, T. Okauchi, N. Ito, D. S. Christovam, A. Marino, D. Takegami, A. Gloskovskii, P. F. S. Rosa, J. Kuneš, S.-I. Fujimori, L. H. Tjeng, A. Severing, A. Hariki, UTe_2: A narrow-band superconductor. *Phys. Rev. Res.* **7**, 043195 (2025).

38. M. Bałanda, AC susceptibility studies of phase transitions and magnetic relaxation: Conventional, molecular and low-dimensional magnets. *Acta Phys. Pol. A.* **124** (6), 964–976 (2013).

39. G. Parisi, *Statistical Field Theory*, vol. 41 of Frontiers in Physics (Addison-Wesley, 1988).

40. A. Arrott, Criterion for ferromagnetism from observations of magnetic isotherms. *Phys. Rev.* **108**, 1394–1396 (1957).

41. A. K. Pramanik, A. Banerjee, Critical behavior at paramagnetic to ferromagnetic phase transition in $Pr_{0.5}Sr_{0.5}MnO_3$: A bulk magnetization study. *Phys. Rev. B* **79**, 214426 (2009).

42. S. T. Bramwell, P. C. W. Holdsworth, Magnetization and universal sub-critical behaviour in two-dimensional XY magnets. *J. Phys. Condens. Matter* **5**, L53–L59 (1993).

43. A. Bedoya-Pinto, J.-R. Ji, A. K. Pandeya, P. Gargiani, M. Valvidares, P. Sessi, J. M. Taylor, F. Radu, K. Chang, S. S. P. Parkin, Intrinsic 2D-XY ferromagnetism in a van der Waals monolayer. *Science* **374**, 616–620 (2021).

44. A. Scheie, M. Ziebel, D. G. Chica, Y. J. Bae, X. Wang, A. I. Kolesnikov, X. Zhu, X. Roy, Spin waves and magnetic exchange Hamiltonian in CrSBr. *Adv. Sci.* **9**, e2202467 (2022).

45. M. E. Fisher, S.-k. Ma, B. G. Nickel, Critical exponents for long-range interactions. *Phys. Rev. Lett.* **29**, 917–920 (1972).

46. P. Chandra, P. Coleman, R. Flint, Hastatic order in the heavy-fermion compound URu2Si2. *Nature* **493**, 621–626 (2013).

47. Y.-f. Yang, Z. Fisk, H.-O. Lee, J. D. Thompson, D. Pines, Scaling the Kondo lattice. *Nature* **454**, 611–613 (2008).

48. N. Nagaosa, J. Sinova, S. Onoda, A. H. MacDonald, N. P. Ong, Anomalous Hall effect. *Rev. Mod. Phys.* **82**, 1539–1592 (2010).

49. K. Schwarz, P. Blaha, Solid state calculations using WIEN2k. *Comput. Mater. Sci.* **28**, 259–273 (2003).

50. J. P. Perdew, K. Burke, M. Ernzerhof, Generalized gradient approximation made simple. *Phys. Rev. Lett.* **77**, 3865–3868 (1996).

51. K. Kim, S. Y. Lim, J.-U. Lee, S. Lee, T. Y. Kim, K. Park, G. S. Jeon, C.-H. Park, J.-G. Park, H. Cheong, Suppression of magnetic ordering in XXZ-type antiferromagnetic monolayer NiPS3. *Nat. Commun.* **10**, 345 (2019).

52. P. Liu, Z. Xu, H. Huang, J. Li, C. Feng, M. Huang, M. Zhu, Z. Wang, Z. Zhang, D. Hou, Y. Lu, B. Xiang, Exploring the magnetic ordering in atomically thin antiferromagnetic $MnPSe_3$ by Raman spectroscopy. *J. Alloys Compd.* **828**, 154432 (2020).

53. K. Binder, P. Hohenberg, Surface effects on magnetic phase transitions. *Phys. Rev. B* **9**, 2194–2214 (1974).

54. K. Binder, "Critical behaviour at surfaces," in *Phase Transitions and Critical Phenomena*, C. Domb, JL Lebowitz, Eds. (Academic Press, 1983), vol. 8, pp. 1–144.

55. X. Guo, W. Liu, J. Schwartz, S. H. Sung, D. Zhang, M. Shimizu, A. L. N. Kondusamy, L. Li, K. Sun, H. Deng, H. O. Jeschke, I. I. Mazin, R. Hovden, B. Lv, L. Zhao, Extraordinary phase transition revealed in a van der Waals antiferromagnet. *Nat. Commun.* **15**, 6472 (2024).

56. G. M. Schmiedeshoff, A. W. Lounsbury, D. J. Luna, S. J. Tracy, A. J. Schramm, S. W. Tozer, V. F. Correa, S. T. Hannahs, T. P. Murphy, E. C. Palm, A. H. Lacerda, S. L. Bud'ko, P. C. Canfield, J. L. Smith, J. C. Lashley, J. C. Cooley, Versatile and compact capacitive dilatometer. *Rev. Sci. Instrum.* **77**, 123907 (2006).

57. C. A. M. dos Santos, A. de Campos, M. S. da Luz, B. D. White, J. J. Neumeier, B. S. de Lima, C. Y. Shigue, Procedure for measuring electrical resistivity of anisotropic materials: A revision of the Montgomery method. *J. Appl. Phys.* **110**, 083703 (2011).

58. J. Xia, P. T. Beyersdorf, M. M. Fejer, A. Kapitulnik, Modified Sagnac interferometer for high-sensitivity magneto-optic measurements at cryogenic temperatures. *Appl. Phys. Lett.* **89**, 062508 (2006).

59. A. Fried, M. Fejer, A. Kapitulnik, A scanning, all-fiber Sagnac interferometer for high resolution magneto-optic measurements at 820 nm. *Rev. Sci. Instrum.* **85**, 103707 (2014).

60. L. C. Chapon, P. Manuel, P. G. Radaelli, C. Benson, L. Perrott, S. Ansell, N. J. Rhodes, D. Raspino, D. Duxbury, E. Spill, J. Norris, Wish: The new powder and single crystal magnetic diffractometer on the second target station. *Neutron News* **22**, 22–25 (2011).

61. O. Arnold, J. C. Bilheux, J. M. Borreguero, A. Buts, S. I. Campbell, L. Chapon, M. Doucet, N. Draper, R. F. Leal, M. A. Gigg, V. E. Lynch, A. Markvardsen, D. J. Mikkelson, R. L. Mikkelson, R. Miller, K. Palmen, P. Parker, G. Passos, T. G. Perring, P. F. Peterson, S. Ren, M. A. Reuter, A. T. Savici, J. W. Taylor, R. J. Taylor, R. Tolchenov, W. Zhou, J. Zikovsky, Mantid—Data analysis and visualization package for neutron scattering and μ SR experiments. *Nucl. Instrum. Methods Phys. Res., Sect. A* **764**, 156–166 (2014).

62. V. Petříček, L. Palatinus, J. Plášil, M. Dušek, Jana2020 – A new version of the crystallographic computing system Jana. *Z. Kristallogr. Cryst. Mater.* **238**, 271–282 (2023).

63. A. J. Freeman, J. P. Desclaux, G. H. Lander, J. Faber, Neutron magnetic form factors of uranium ions. *Phys. Rev. B* **13**, 1168–1176 (1976).
